# Exploitation of fibrin-based signaling niche for deriving progenitors from human adipose-derived mesenchymal stem cells towards potential neural engineering applications

**DOI:** 10.1038/s41598-020-63445-2

**Published:** 2020-04-28

**Authors:** Krishnapriya Chandrababu, Manesh Senan, Lissy K. Krishnan

**Affiliations:** 1 Division of Thrombosis Research, Department of Applied Biology, Biomedical Technology Wing, Sree Chitra Tirunal Institute for Medical Sciences and Technology, Thiruvananthapuram, Kerala 695012 India; 2 Department of Plastic Surgery, Kerala Institute of Medical Sciences (KIMS), Thiruvananthapuram, Kerala 695 029 India

**Keywords:** Reverse transcription polymerase chain reaction, Regenerative medicine

## Abstract

Adipose-derived mesenchymal stem cells (hADMSC) retaining proliferation and multi-differentiation potential may support the central nervous system (CNS) regeneration. Multipotency of MSC may result in both desirable and undesirable cells, post-transplantation. A better strategy to attain desired cells may be *in vitro* commitment of hADMSCs to uni-/bi- potent neural progenitor cells (NPCs), prior to transplantation. Derivation of stable NPCs may require a suitable niche eliciting proliferation and differentiation signals. The present study designed a biomimetic niche comprising insoluble fibrin supported adhesion matrix and exogenously added growth factors (GFs) for deriving different neural cells and established the role of Notch and Wnt signals for proliferation and differentiation of hADMSCs, respectively. The stable transformation of hADMSCs into neurospheres (NS) comprising Nestin^+ve^ NPCs was achieved consistently. Slight modifications of niche enable differentiation of NS to NPCs; NPCs to neurons; NPCs to oligodendrocyte progenitor cells (OPCs); and OPCs to oligodendrocytes (OLG). Fibrin plays a crucial role in the conversion of hADMSC to NS and NPCs to OPCs; but, not essential for OPC to OLG maturation. Co-survival and cell-cell interaction of NPC derived neurons and OPCs promoting OLG maturation is illustrated. The designed biomimetic niche shows the potential for directing autologous ADMSCs to neural cells for applications in regenerative medicine.

## Introduction

Stem cells can promote the regeneration of tissues either by substitution of the lost cells or by providing the trophic support, homing and differentiation to proliferating progenitors or terminally differentiated functional cells at the injury site. The human adipose-derived mesenchymal stem cells (hADMSC) are widely recognized for applications in regenerative medicine^1^. The multipotency of hADMSC promises the opportunity for producing diverse progenitors and functional cell types resulting in the regeneration of diseased tissues. However, like a double-edged sword, multipotency could be a weakness, as they may differentiate *in vivo* to undesirable lineages causing the adverse outcome. The lack of adequate signals in the injured and degenerating hostile tissue may not always direct MSCs to desired differentiation. Therefore, the differentiation of hADMSCs into required cell lineages, prior to transplantation may be considered a better strategy to improve therapeutic outcomes. The terminal differentiation of progenitors to functional cells inversely affects proliferation which in turn could reduce the regeneration potential *in vivo*^2^. Hence, achieving a controlled process of lineage commitment to the progenitor stage, which could maintain a balance of proliferation and differentiation potential is a challenging problem to address.

The injured central nervous system (CNS) tissue shows limited and slothful ability to regenerate. This may be because of the inadequate number of endogenous neural progenitor cells (NPC) and the development of unfavorable micro-environment post-injury, affecting cell homing and differentiation. The stem cell transplantation is considered as a prospective therapy for CNS injury, considering the trophic support and its potential to differentiate into specific cells in the injured region^3^. The injury-associated and other degenerating diseases affecting CNS require glial and neuronal cells for its regeneration into fully functional tissue. Therefore, the exploitation of multi-potent hADMSCs may aim generation of NPCs and oligodendrocyte progenitor cell (OPC) with proliferation/differentiation potential starting from the same source of hADMSC, for mixed cell transplantation. Differentiation into each cell type may require specific niche conditions consisting of adhesive protein and growth factors (GF).

Several advantages of the fibrin-based niche in stem cell growth and differentiation have been reported. Polymerized fibrin network constitutes several other adhesive proteins including fibronectin(FN) and laminin(La) playing a prominent role in eliciting signals for proliferation, survival, and differentiation. A previous study reported that both FN and La are responsible for stimulating Nestin^+ve^ progenitors in peripheral blood mononuclear cells (PBMNC) directing differentiation to neurons^4,5^. The heparin-binding domains of the FN can immobilize GFs like PDGF, FGF, TGF-β, and neurotrophin families of GFs^6^. Thus, fibrin may be considered as a GF reservoir and producing various signals based on the GFs added into the medium for specific cellular effects. Various *in vitro* studies established that the fibrin-based niche is efficient in promoting differentiation and proliferation of stem/progenitor cells to neurons, keratinocytes or endothelial cells^5,7–9^. The established role of the human fibrin-based composite niche for selective adhesion of NPCs instigated the exploration of hADMSC differentiation to neural cells. Neurogenic signals in the fibrin niche may promote stable differentiation, unlike the transient changes that have been often described^10,11^. Also, most of the protocols described for pre-differentiating hADMSCs take a longer time in culture^12,13^. A reduction in the culture period would be highly beneficial in clinical translation. Therefore, this study attempted cell-specific modification of fibrin-based niche to obtain stage-wise and stable differentiation of hADMSCs to both neural and glial cells. The control of differentiation of mesodermal cells to ectodermal cells through various minor alterations of the niche was the primary objective of the study. Only established biochemical pathways could cause step-wise and steadily progressing stable progenitors; therefore, the role of two important biomimetic signaling pathways was studied. Since differentiated neurons or oligodendrocytes are not suitable for effective transplantation therapy, a functional assay of the differentiated cells is beyond the scope of this study. However, the differentiation potential of NPCs to oligodendrocytes and neurons were established using multiple markers.

## Results

### Qualification of hADMSC

The isolated hADMSCs showed typical stem cell properties in terms of surface marker expression and trilineage differentiation potential. The results are presented in the Supplementary File. The tri-lineage differentiation potential and classical MSC surface marker expressions are in accordance with the standards recommended by the International Society for Cellular Therapy (ISCT). The isolation protocol was found suitable for obtaining pure hADMSCs with good proliferation potential and multipotency meeting the pre-requisites for *in vitro* differentiation to neural lineage cells.

### Fibrin based niche in ADMSCs to NS conversion

**Figure 1:**
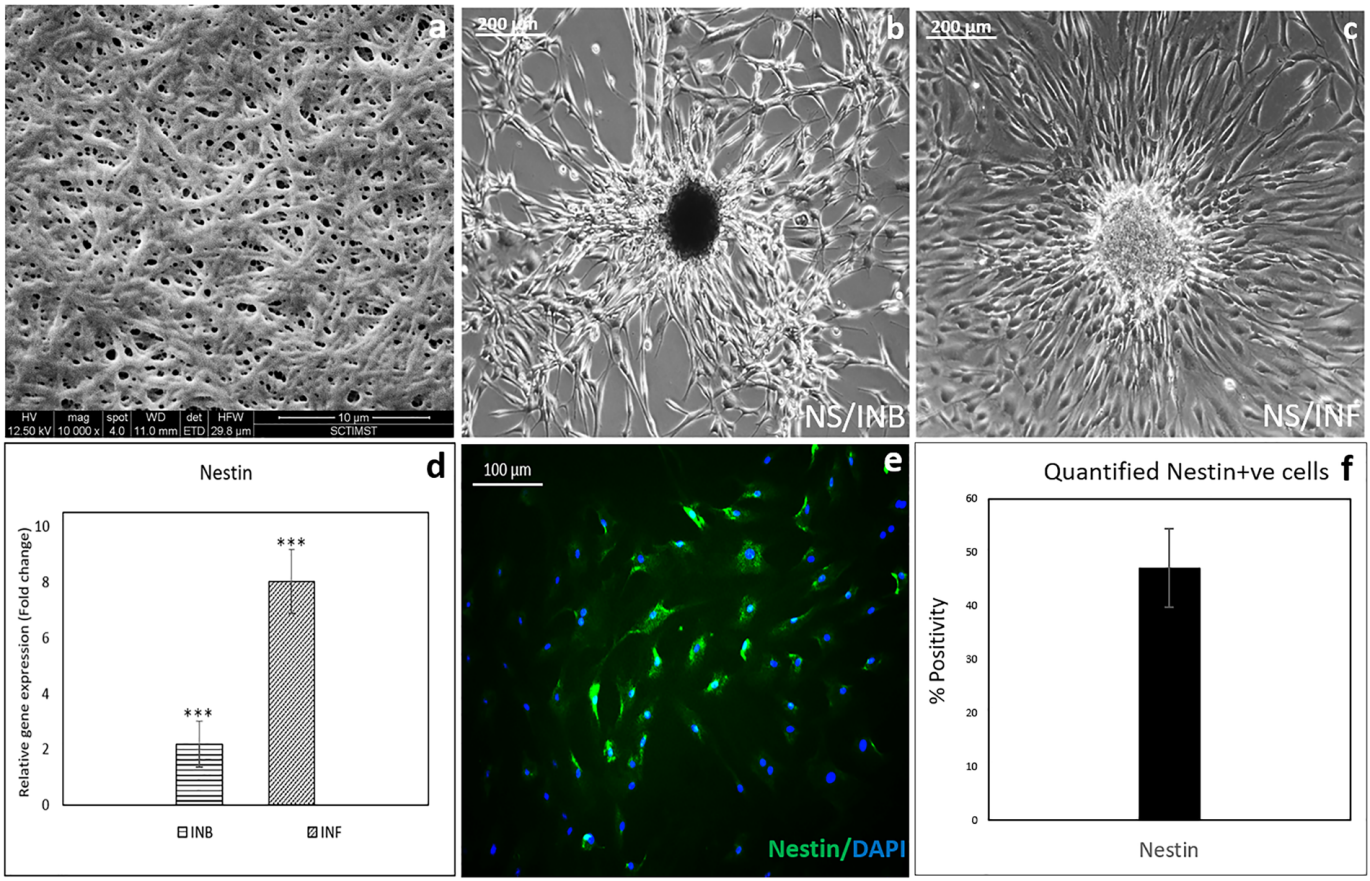
Characteristics of hADMSC derived NS: Phase-contrast Micrographs of NS derived from hADMSCs. (**a**) Fibrin niche fiber morphology and porosity (10,000x magnification); (**b**) Mature NS by day 7 in bare TCPS (INB); (**c**) Mature NS by day 7 in fibrin niche (INF); (**d**) Graphical representation showing qRT-PCR data of Nestin gene expression relative to hADMSCs; (**e**) Fluorescent micrograph of NS cells immunostained with Nestin antibody by day 7 on INF (periphery showing isolated cells); (**f**) The graphical representation of Flow cytometric analysis data of Nestin in 3 donor cells. For qRTPCR, hADMSC in bare TCPS grown in DMEM F12 media for 7 days was used as the experimental control; GAPDH was used as the Housekeeping gene; ANOVA: Control, INB & INF; P = 0.001(n = 3); ‘***’ (P ≤ 0.001),‘**’ (P ≤ 0.01),‘*’ (P ≤ 0.05); Error bar represents SEM.

The fibrin matrix coated on tissue culture polystyrene (TCPS) showed fibrous and porous morphology (Fig. 1a). The fibers appear thick and suitable for cell adhesion allowing its spreading to establish good contact with the biomolecules present in the matrix. The chance of seeded cells contacting the tissue culture polystyrene (TCPS) surface seemed doubtful because of the uniform spreading of fibrin mesh covering the polystyrene surface. Therefore, the behavioral difference between hADMSCs grown on bare TCPS and fibrin may be attributed to the property of the latter. The effect seen in TCPS is mainly due to the signaling by GFs added in the induction medium (IM). The hADMSC cultures grown on bare TCPS and induced by GFs supplemented in the IM is termed as INB. The hADMSC cultures grown on fibrin coated TCPS in the presence of IM are termed INF.

The hADMSCs grown in both INB and INF became thin with increased cell-cell contact by day 4, when the seeding density was 10000 cells per cm^2^ (Fig. 1b,c). The hADMSC seemed to proliferate and developed groups of cells which transformed further into distinct spheres denoted as neurosphere (NS) in 7 days in both INB and INF (Fig. 1b,c) cultures. The NS adherence appeared similar in both INB and INF conditions, at the early stage of induction, with noticeably better anchoring in INF when observed under a phase-contrast microscope. Most of the NS in INB appeared dark under the microscope in a time period between 7 to 10 days and detached from the dish suggesting cell death. The NS in INF were stable, bright, healthy and alive for >10 days. The preliminary appearance of the NS growing in INF and the cells migrating out from the NS suggested better stability (Fig. 1c). Also, it was noted that when 5000 hADMSCs per cm^2^ was seeded, both INF and INB was not able to form NS consistently (not shown), indicating cell density is important for grouping and NS formation.

The INF showcased more neural lineage conversion as evident from the qRT-PCR data, displaying significant up-regulation of Nestin by the 7^th^ day of culture (Fig. 1d). The qualitative analysis of immunostained NS cells in the niche, using fluorescence microscopy revealed the presence of Nestin in the NS grown on INF (Fig. 1e). In addition to qualitative analysis, the quantitative analysis using flow cytometry of Nestin stained cells also confirmed that ~45–50% cells in NS developed on INF were Nestin^+ve^ (Fig. 1f). These preliminary results indicate the advantage of growing hADMSC for inducing into NS resulting in the upregulation of NPC marker, Nestin- both at the transcriptional and translational level. Therefore, NS picked from INF was used for further evaluations.

### Growth of secondary NS

**Figure 2:**
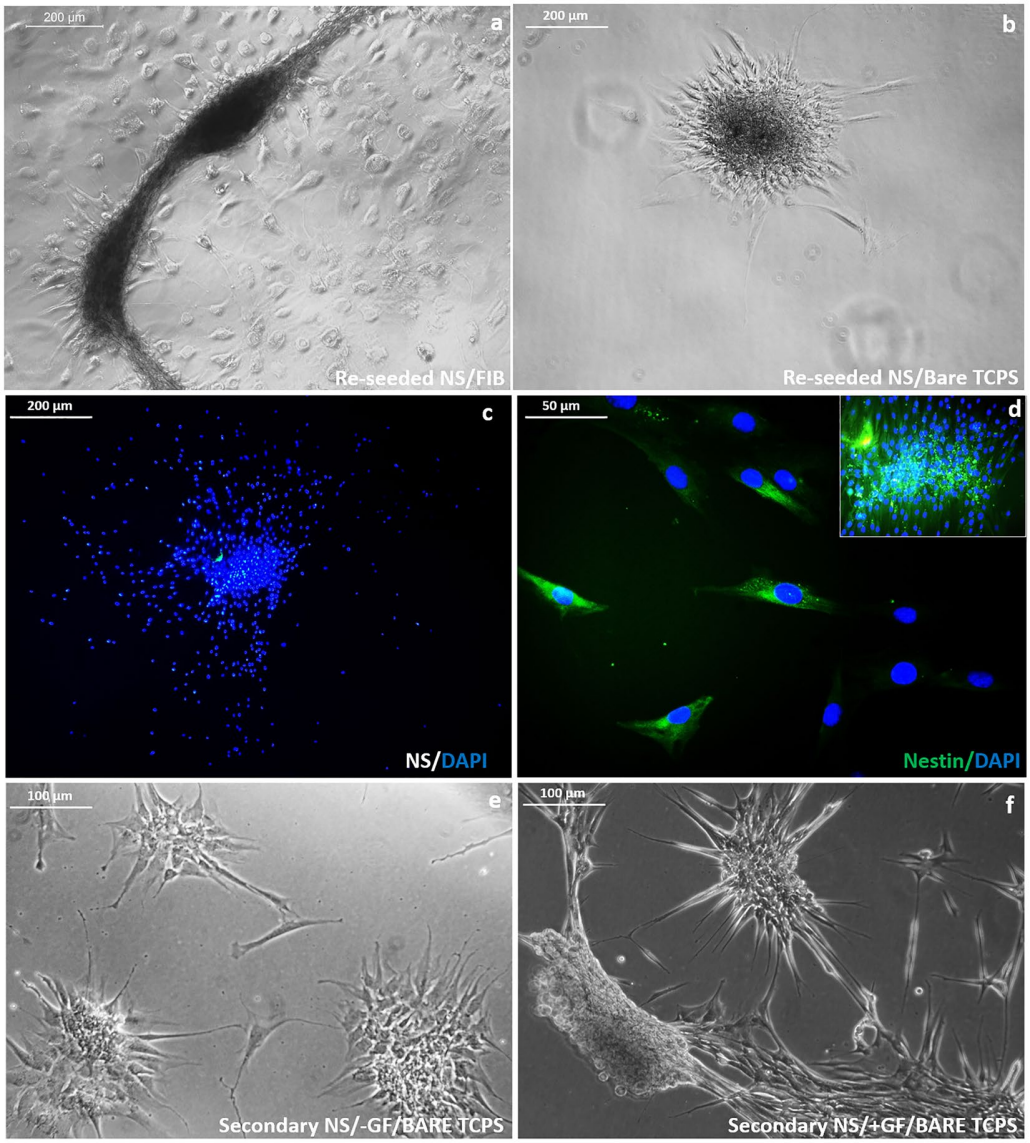
Secondary sphere formation potential of Nestin positive NS cells. (**a**) Phase contrast micrograph showing NS reseeded by manual picking in (**a**) fibrin coated TCPS; (**b**) bare TCPS; (**c**) DAPI stained cell nucleus of reseeded NS in fibrin matrix; (**d**) Fluorescent micrograph showing reseeded NS immunostained in INF for Nestin- counterstained with DAPI at 40x magnification with inset 10x magnification; (**e**) Secondary NS formation by day 5 on bare TCPS in normal medium without GF; (**f**) Morphology of secondary NS by day 5 in bare TCPS with IM.

Manually picked NS derived on INF, attached similarly on both fibrin-TCPS and bare-TCPS in 24 h (Fig. 2a,b). Within 5days, the attached NS cells sprouted out and became confluent in fibrin dish covering about 1.75 cm^2^ area (not shown). Nuclear staining using DAPI indicated the presence of numerous nucleus within the reseeded and grown NS (Fig. 2c). Many cells in and around the propagating NS immunostained positive for Nestin (Fig. 2d).

The trypsin-dissociated primary NS seeded on bare TCPS and fibrin-TCPS formed secondary spheres in the absence (Fig. 2e) and presence (Fig. 2f) of added GFs in the medium. Some of the secondary spheres formed were bigger and visible by the naked eye while others were smaller & visible under the microscope only (not shown). The results indicate maintenance of stem-ness and proliferation potential even after trypsinization of the NS. Poor long-term stability and detachment of secondary NS grown in INB were evident; therefore, INF can be considered as a better strategy to maintain the secondary NS in the culture.

### Proof for Wnt & Notch pathways during hADMSCs to NS conversion on INF

**Figure 3:**
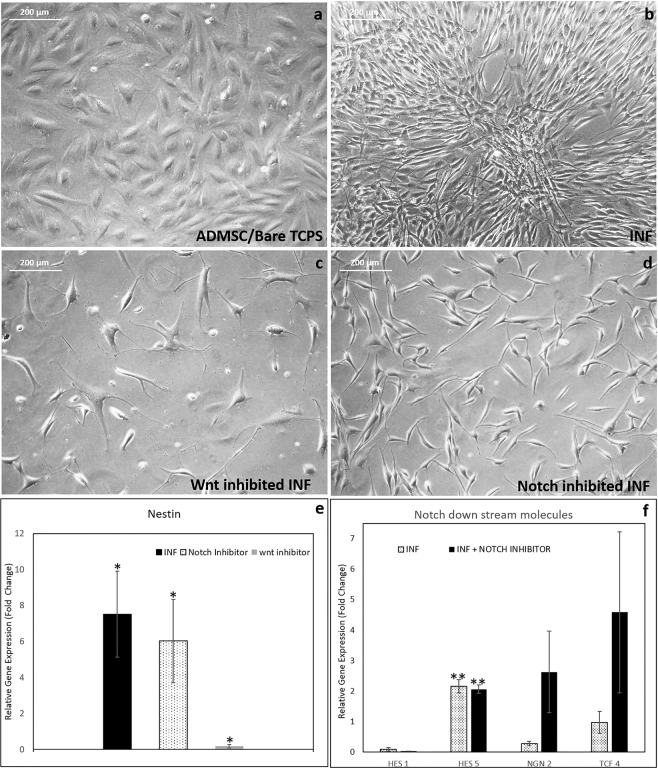
Analysis of notch and wnt signaling inhibition in NS formation by day 5. (**a**) Phase-contrast micrograph of normal proliferating hADMSC cultured on bare TCPS used as the experimental control; (**b**) Phase-contrast micrograph of hADMSCs induced to NS in fibrin niche (INF) showing grouping; (**c**) Morphology of wnt inhibited INF cultures; (**d**) Morphology of notch inhibited INF culture; (**e**) Graphical representation of qRT-PCR analysis of Nestin marker in INF (T1) and both wnt- & notch- inhibited INF; (**f**) Graphical representation of qRT-PCR analysis of notch downstream markers, HES-1, HES-5, Ngn-2, and TCF-4. For all qRT-PCR, hADMSCs in bare TCPS was used as experimental control; GAPDH was used as Housekeeping gene;ANOVA: Control, INF, Notch inhibited INF; Hes 5, P = 0.002; n = 3; (‘*’ (P < 0.05) ‘**’ (P < 0.01) ‘***’ (P < 0.001)).

Cell proliferation and cell density were found to be important for NS formation on INF. Therefore, two signaling cascades-Notch & Wnt- responsible for neural stem cell (NSC) proliferation and differentiation were explored as possible pathways prominent in INF. Normal proliferating hADMSC cultured on bare TCPS was used as the experimental control-C (Fig. 3a) to study the upregulation of both Wnt/Notch signals on INF (T1). Using chemical inhibited (Wnt inhibitor/Notch inhibitor) INF cultures (T2), differential marker expressions among C, T1 and T2 assessed by statistical methods (ANOVA) is illustrated as proof to distinguish involvement of Wnt and Notch signaling in INF. The INF cells showed grouping after 5 days of seeding (Fig. 3b). The cell density in Wnt inhibited culture was lower with minimal cell to cell contact as compared to INF, indicating a major role of Wnt in inducing cell proliferation on fibrin (Fig. 3c). When mRNA was isolated from the remaining cells in Wnt inhibited cultures, significant down regulation of Nestin was observed suggesting inhibition of differentiation by Wnt inhibitor (Fig. 3e).

Two different concentrations of the Wnt inhibitor evaluated its dose-response on stage-specific marker protein expression at the translational level. Secreted Wnt 3a is expressed prominently in ECM of INF grown cells as compared to that in control ADMSC (Supplementary Fig. 2a,b). Both low (LC −20 μM/ml) and high inhibitor concentrations (HC-40 μM/ml) reduced Wnt molecules in the ECM of the cells prominently (Supplementary Fig. 2c,d). The β-catenin molecules which were visualized in the nucleus of induced cells also indicated active Wnt signaling (Supplementary Fig. 3a). The prominent presence of Wnt-3a in ECM and β-catenin molecules in the nucleus was rarely noticed in control hADMSC cultures (Supplementary Figs. 2b and 3b), suggesting minimal activation of Wnt signals in control. The lower expressions of Wnt-3a and β-catenin in the inhibited cultures as compared to INF suggested that active Wnt signaling is operational in the fibrin-based niche. Significantly down-regulated Wnt in inhibited INF in parallel to reduced Nestin expression indicates differentiation of hADMSC to NPCs in NS involves β-catenin mediated Wnt signaling.

The cells in Notch inhibited INF appeared to be thin and elongated similar to that of neural cell morphology (Fig. 3d). The Notch inhibited cultures-T2 hardly developed any cell groups with reduced cell to cell contact. The expression of Nestin in the available cells growing in the Notch inhibited culture was seen upregulated and similar to that in INF (Fig. 3e). This observation suggests that Notch signaling is important in cell proliferation; but, even when Notch is inhibited, the hADMSC to NPCs differentiation proceeded. Upon adding 20 uM ml^−1^ of Notch inhibitor into the culture, upregulation of the downstream signaling molecules Ngn-2 by ~2.6 fold and TCF-4 by ~4.5 at mRNA level was seen (Fig. 3f). The Hes-1 gene expression remained quiescent or unchanged in both INF and inhibited INF conditions. Another downstream molecule Hes-5 is significantly upregulated similarly in both in INF and inhibited INF.

The Notch molecules were prominent in the nucleus indicating active Notch signaling in the niche (Supplementary Fig. 4a). The control ADMSCs in bare TCPS also showed active Notch molecules in the nucleus (Supplementary Fig. 4b). Some active Notch molecules were seen in Notch inhibited cultures even with a higher concentration (HC) of inhibitors (20 μM/ml-LC; and HC 40 μM/ml) (Supplementary Fig. 4c,d). Complete inhibition was not achieved even with HC of inhibitor. Therefore, the prominent role of Notch in INF advocates higher cell proliferation. No specific role of Notch signals from fibrin is indicated for differentiation because of Nestin upregulationis illustrated even in Notch inhibited cultures (Fig. 3d).

### Progression of NPCs upon growing on INF

**Figure 4:**
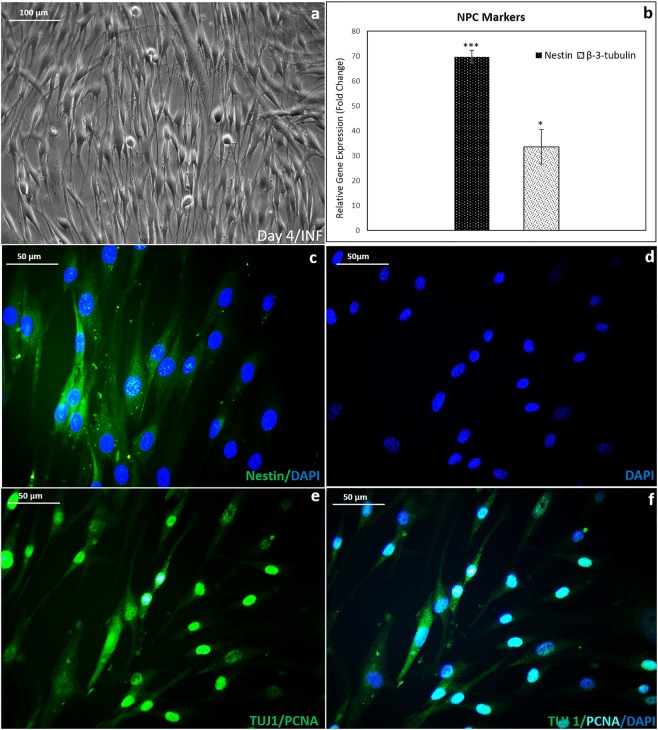
NPC morphology and gene expression in fibrin niche. (**a**) Phase-contrast micrograph of cells after 4 days of NPC progression; (**b**) Graphical representation of qRT-PCR analysis of NPC markers, Nestin and TUJ-1 (β-3-tubulin); (**c**) Immunostained Nestin marker on day 4, DAPI represents nucleus; (**d**) DAPI stained NPC nucleus; (**e**) fluorescence micrograph of NPC stained with PCNA & TUJ -1 antibodies, PCNA in nucleus of proliferating cell and TUJ-1, tubulin protein in the cytoplasm; (**f**) PCNA and DAPI co-localized in nucleus as cyan color and TUJ-1 seen as green. For estimating relative gene expression, hADMSC in bare TCPS in DMEM F12 media for 7 days was used as the experimental control; GAPDH was used as the Housekeeping gene; Student’s t-test: Control & NPC in niche; Nestin: P = 0.001 (n = 3); β-3-tubulin: P = 0.05 (n = 3); ‘***’ (P ≤ 0.001),‘**’ (P ≤ 0.01),‘*’ P ≤ 0.05; Error bars represent SEM.

Upon adding IM, the dissociated NS from INF seeded on fibrin showed thin elongated morphology within 4 days (Fig. 4a). The specific markers, Nestin and TUJ-1/(Beta-3-tubulin) showed upregulation in the NS-derived NPCs upon qPCR analysis (Fig. 4b). Fold change (relative gene expression) >70 was observed for the Nestin marker, while fold change >30 was obtained for TUJ-1 in induced NPC-like cells. The high abundance of Nestin enabled western blot analysis confirming nestin^+ve^ in the induced cells. Bands at ~260 kDa and ~120 kDa were observed (Supplementary Fig. 5).

The maintenance of NPCs in the culture was confirmed by immunostaining for Nestin (Fig. 4c). The proliferation of NPCs in the culture was established by PCNA-TUJ-1 dual-immunostaining (Fig. 4d-f). Both these immuno-markers were found localized to specific cell compartments. The green cytoplasm and cyan nucleus indicate that the NPCs grown on fibrin-TCPS are proliferating well. The TUJ-1^−ve^ proliferating cells could be the hADMSCs that have not undergone differentiation; therefore, protein isolates were not suitable for getting good quality WB. The flow cytometric analysis showed ~30% cells to be TUJ-1^+ve^ (data not shown).

### Conversion of NPCs to OPCs on INF

**Figure 5:**
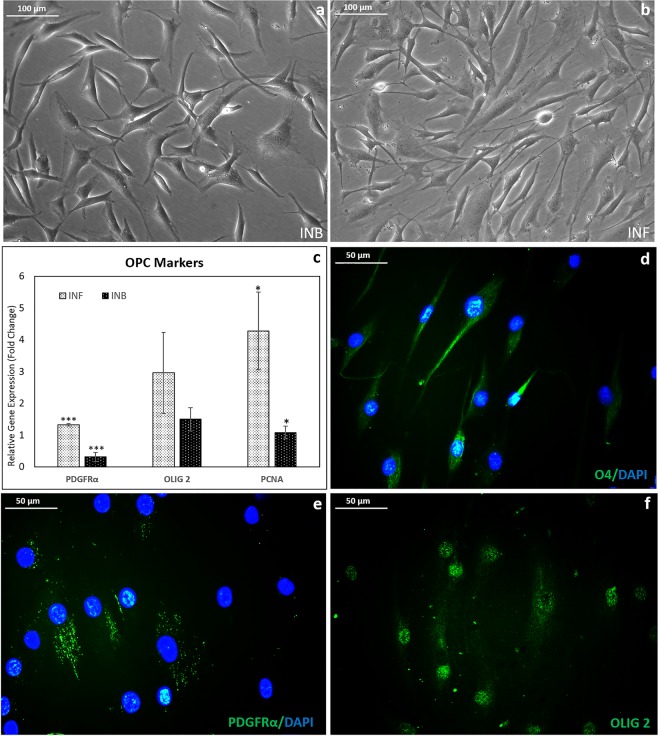
Analysis of OPC marker expression in induced cultures in niche: Phase-contrast micrograph of OPC induced from NPCs by day 4 (**a**) in bare TCPS (INB); (**b**) in fibrin niche (INF); (**c**) Graphical representation of qRT-PCR analysis of proliferation marker PCNA and OPC markers PDGFRα & OLIG-2; (**d**) fluorescence micrograph showing O4 antigen stained cells growing on INF; (**e**) Immunostained PDGFRα^+ve^ OPCs growing on INF; (**f**) OLIG-2 nuclear specific marker stained OPCs growing on INF-(in this the nuclear stain, DAPI was not used). Relative gene expression profile of NPCs induced to OPCs was estimated using hADMSC in bare TCPS in DMEM F12 media as experimental control; ANOVA: Control, INB & INF; PDGFR-α, P = 0.0004 (n = 3), PCNA, P = 0.02 (n = 3). GAPDH used as the Housekeeping gene; (‘***’ (P ≤ 0.001),‘**’ (P ≤ 0.01),‘*’ P ≤ 0.05)); Error bars represent SEM.

Transformation into OPC-like morphology was evident when the INF-derived NPCs were further grown in the presence of specific GF in both INB (Fig. 5a) and INF (Fig. 5b). On INF, more OPC-like phase bright cells were observed. One specific marker OLIG-2 was slightly upregulated on INB; however, PDGFRα was down-regulated with respect to hADMSC control (Fig. 5c). On INF, both OLIG-2 and PDGFRα were upregulated suggesting the progression of OPC phenotype upon induction on fibrin matrix (Fig. 5c). The significant upregulation of the PCNA gene indicated the proliferation potential of induced OPCs on INF (Fig. 5c). The PDGFRα and PCNA gene expressions were lower in INB than the hADMSC control indicating poor proliferation potential of OLIG-2 positive cells on INB (Fig. 5c).

In cells on INF culture, O4 antigen, OLIG-2, and PDGFRα markers were prominently expressed upon immunostaining (Fig. 5d–f). The O4 and PDGFRα molecules were observed in the cell membrane, while the OLIG-2 transcription factor was observed in the nucleus of the induced cells. Quantitative flow cytometric analysis data of OLIG- 2^+ve^ OPCs showed ~30% population upon replicate donor cell experiments (n = 3) (Supplementary Fig. 6a–d). The western blotting confirmed the presence of OLIG-2 protein in the induced cells. A ~36 kDa protein band was observed (Supplementary Fig. 6e)

### Proof of Wnt and Notch signaling pathways in NPCs to OPCs conversion on INF

**Figure 6:**
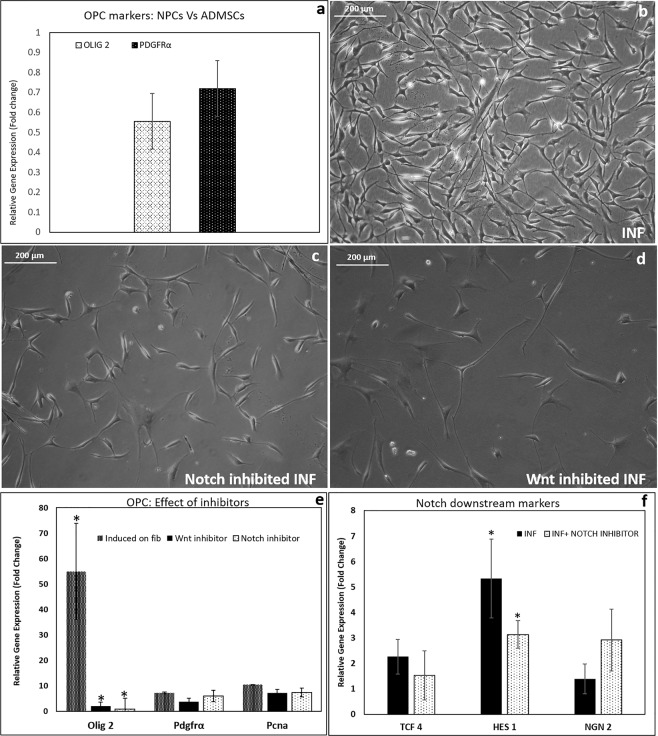
Effect of notch and wnt signal inhibitors upon OPC induction in INF. (**a**) Relative gene expressions of OPC markers (OLIG-2 & PDGFRα) in NPCs as compared to proliferating hADMSC cultured on bare TCPS estimated using qRT-PCR. hADMSC grown in bare TCPS used as experimental control and GAPDH used as Housekeeping gene; (**b**) NPCs induced in fibrin niche showing OPC like morphology by day 4; (**c**) Morphology of notch inhibited INF culture on day 4; (**d**) Morphology of wnt inhibited INF culture on day 4; (**e**) Graphical representation of qRT-PCR analysis of markers, OLIG-2, PDGFRα and PCNA; (**f**) Graphical representation of qRT-PCR analysis of notch downstream markers after 4 days of induction; ADMSC grown in bare TCPS used as experimental control and GAPDH used as Housekeeping gene;ANOVA: INF, Wnt inhibitor & Notch inhibitor; OLIG- 2: P = 0.02 (n = 3); HES-1: P = 0.04(n = 3); ‘*’ (P < 0.05) ‘**’ (P < 0.01) ‘***’ (P < 0.001).

The OPC markers (OLIG-2 & PDGFRα) were not upregulated in starting NPCs as compared to proliferating hADMSC cultured on bare TCPS (Fig. 6a). Therefore, hADMSC was used as the experimental control-C to estimate the upregulation of markers. Upon induction, the cells showed OPC like morphology in INF (T1) within 4 days (Fig. 6b). The cells with OPC morphology was present in Notch inhibited (T2) culture also (Fig. 6c). However, OPC-like morphology was rarely seen in Wnt inhibited culture (T2)(Fig. 6d). The cell density in Wnt inhibited culture was very low and showed neural-like thin elongated morphology.

Though OPC-morphology was seen in Notch inhibited culture, the qRT-PCR analysis showed significant down-regulation of OLIG-2gene expression as compared to OPCs in INF (Fig. 6e). In Wnt inhibited cultures, the downregulation of OLIG-2 correlated well with the absence of OPC morphology (Fig. 6d). The upregulation of PDGFRα and PCNA gene expression showed a significant fold increase in INF. The Wnt inhibited cultures down-regulated PDGFRα gene expression more than that of Notch inhibited cultures (Fig. 6e). Therefore, Notch inhibition is considered less effective in preventing NPC to OPC differentiation on INF.

The qRT-PCR analysis of the cells induced to OPCs in Notch inhibited INF (20 μM/ml) changed the extent of gene expression of various downstream markers when compared to that of INF (Fig. 6f). The pattern of Ngn-2 gene expression in INF cells indicated active Notch signaling in the culture. Hes-1 expression was significantly down-regulated in inhibited culture as compared to INF cells. This also confirmed the presence of active Notch signaling in the niche during OPC induction. The TCF-4 expression was upregulated similarly in both INF and inhibited conditions. The results suggest that the inhibitors have not been able to block the differentiation. However, the role of Notch signaling seems to be important for differentiating NPCs to proliferating OPCs on INF.

The co-localization of Notch receptor & DAPI in the nucleus of induced cells were indicative of active Notch signaling in the INF culture. The Notch molecules were more prominent in cells grown on INF with intensely stained nucleus (Supplementary Fig. 7a). Control hADMSC grown in bare TCPS showed active Notch molecules in the nucleus but comparatively lower numbers were seen (Supplementary Fig. 7b). The active Notch molecules in the nucleus were seen in cells treated with both concentrations of inhibitor (10 μM/ml; LC and 20 μM/ml; HC; Supplementary Fig. 7c,d) similar to that in control hADMSC. However, the OLIG-2 down-regulation in the inhibited culture and morphology together indicates that the signaling was not sufficient for the conversion of NPCs to the OPC phenotype.

Immunostaining of Wnt molecules in the INF showed prominent expression of these molecules in the ECM (Supplementary Fig. 8a). Both control hADMSC and Inhibitor added INF showed Wnt molecules in the cytoplasm but not in ECM (Supplementary Fig. 8b–d). Results indicate that complete inhibition of Wnt signals was achieved using the inhibitor. Immunostaining of induced OPC cells using the β-catenin antibody showed prominent expression of β-catenin in the nucleus of INF cells (Supplementary Fig. 9a). The active beta-catenin molecules were observed in a few of the immunostained hADMSC controls (Supplementary Fig. 9b). In the Wnt inhibited INF cultures, characteristic stains of active β-catenin molecules were hardly seen in most of the nucleus (Supplementary Fig. 9c,d). Therefore, Wnt inhibition adversely affected OPC morphology (Fig. 6d) and downregulated the expression of OLIG-2 (Fig. 6e).

### Fibrin directed differentiation of NPCs to neurons

**Figure 7:**
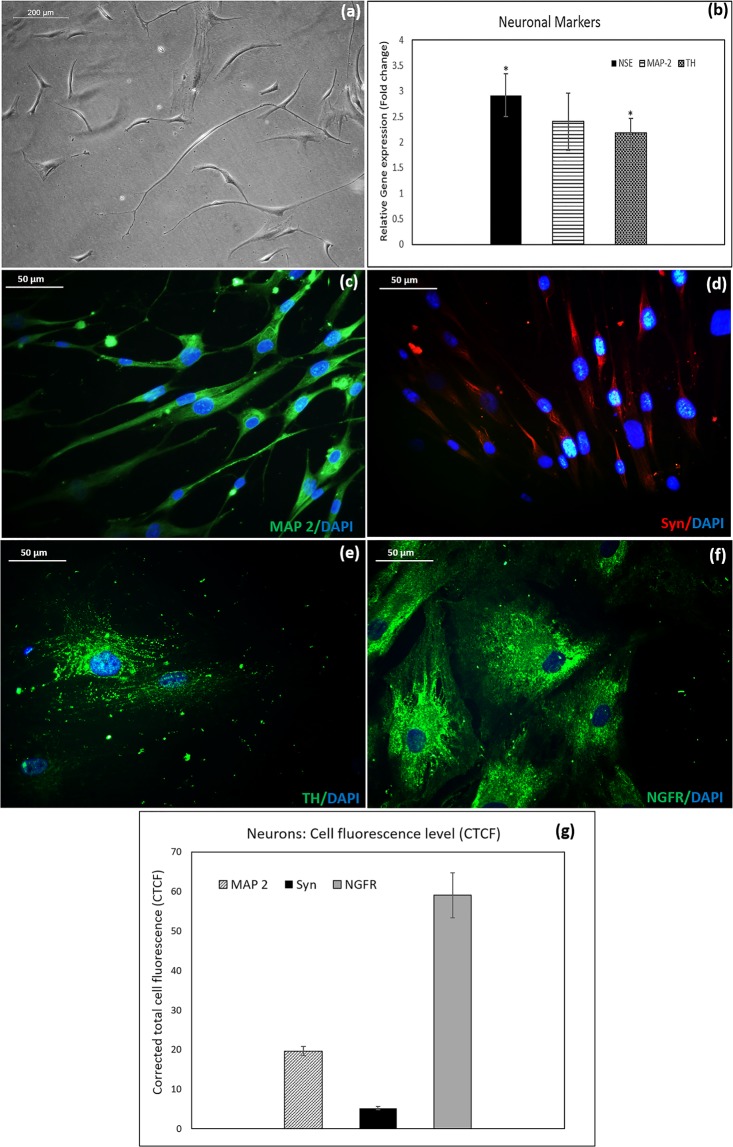
Illustration of neurons derived from hADMSC-NPCs. Morphology and specific marker expression of the induced neurons: (**a**) The phase-contrast micrograph showing elongated neural morphology of cells induced for 6 days; (**b**) Graphical representation of qRT-PCR analysis of neural markers: NSE, MAP-2 and TH gene expression compared to control ADMSCs. Mature neuronal markers NSE (P = 0.05) and TH (P = 0.04) showed significant upregulation of gene expression when compared to control cells. Student’s t-test: Control & INF used for analysis. hADMSC in bare TCPS in DMEM F12 media for 7 days was used as the experimental control; GAPDH was used as the Housekeeping gene; ‘***’ P ≤ 0.001, ‘**’ P ≤ 0.01, ‘*’ P ≤ 0.05; Error bars represent SEM. The fluorescence micrograph showing mature neuronal markers in induced cells: (**c**) MAP 2; (**d**) Syn (**e**) TH (Dopaminergic neuron marker); (**f**) NGFR. DAPI used as nuclear stain. (**g**) Quantitative fluorescence image analysis: Corrected total cell fluorescence (CTCF) calculated from ICC images of MAP 2, Syn and NGFR (Average of CTCF of 15 cells from 5 fields). Error bars represent SEM.

NPCs upon neuronal induction showed thin elongated cell morphology. The neural extensions were more prominent after the addition of KCl to the niche (Fig. 7a). The mature neuronal marker expression at the mRNA level was significantly upregulated in the induced culture by day 6 (Fig. 7b). The neuron-specific cytoskeletal protein-microtubule associated protein −2 (MAP-2) was found to be upregulated at the mRNA level in the induced neurons. The gene expression of neuron-specific enolase (NSE) was significantly upregulated in the induced culture as compared to the control hADMSCs. The dopaminergic neuronal marker tyrosine hydroxylase (TH) was significantly upregulated in the induced cells indicating terminal differentiation of NPCs to dopaminergic neurons also. The findings from the qRT-PCR analysis were further established at the protein level by immunostaining of induced neuronal cells against MAP-2 antibody (Fig. 7c), Synaptophysin (Syn) antibody (Fig. 7d), TH antibody (Fig. 7e) and Nerve growth factor Receptor (NGFR) antibody (Fig. 7f). The expression of 4 different immune-markers confirms the differentiation of NPCs to neurons.

### Conversion of OPCs to OLG in INF & INB

**Figure 8:**
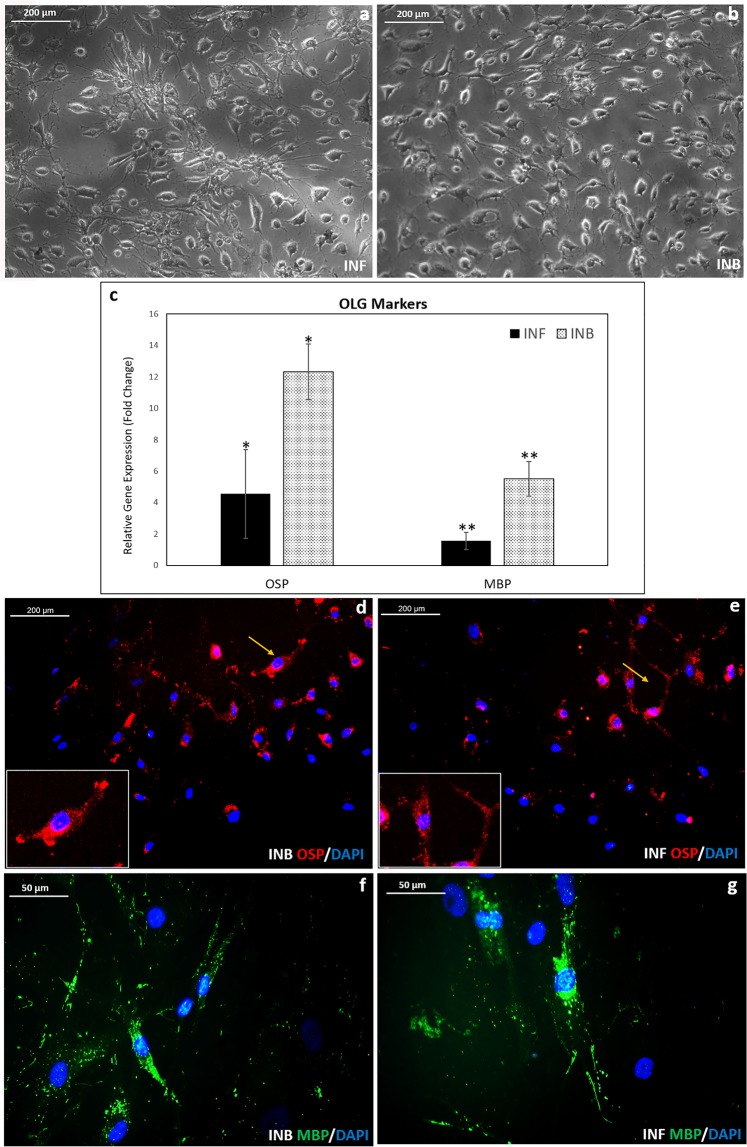
Analysis of induced OLGs in the niche and bare TCPS. (**a**) Phase contrast micrograph showing morphology of induced OLGs in fibrin niche (INF) by day 2; (**b**) morphology of induced OLGs in bare TCPS (INB) by day 2; (**c**) Graphical representation of qRT-PCR analysis of OLG markers: ANOVA: Control, INB & INF; OSP: P = 0.015 (n = 3) MBP: P = 0.008 (n = 3); hADMSC in bare TCPS in DMEM F12 media for 7 days used as the experimental control; GAPDH used as the Housekeeping gene; (‘***’ (P ≤ 0.001),‘**’ (P ≤ 0.01),‘*’ P ≤ 0.05)); Error bars represent mean SEM; (**d**) Fluorescence micrograph showing immunostained OLG markers by day 2 on INB (**e**) OSP immunostained OLGs in INF (magnified region of interest in fluorescence micrograph shown as inset);(**f**) MBP stained induced OLGs in bare TCPS; (**g**) MBP stained induced OLGs in fibrin niche. DAPI used as nuclear stain.

After 48 h induction, the OPCs showed dendrites like projection similar to OLGs in both fibrin matrix and bare TCPS (Fig. 8a,b). Cells with OLG morphology seemed to detach from the cell culture dishes in INB and INF.

Gene expression analysis of the induced OLGs show upregulated expression of mature OLG markers, oligodendrocyte specific protein (OSP) and myelin basic protein (MBP) in both INB and INF (Fig. 8c). Significant upregulation of both OSP and MBP was observed in bare TCPS. However, both genes showed marked upregulation in INF also, as compared to control. The expression of OLG markers was confirmed at the translation level by staining the induced cells with MBP and OSP antibodies in both INB and INF conditions. Qualitatively, the protein expression appeared similar in both the bare TCPS and fibrin niche (Fig. 8d–g).

### Co-survival of OPCs and neuron in the fibrin matrix

The cells with OLG morphology appeared in the culture after 3 days of co-culture of neurons and OPCs in the presence of KCl added basal DMEM F12 media (Supplementary Fig. 10a). The gene expression analysis of OLG and neuronal markers using qRT-PCR showed upregulation of neuronal markers TUJ-1, MAP-2 and OLG marker OSP at the mRNA level. Glial fibrillary protein (GFAP), an astrocyte-specific marker was significantly upregulated in the mRNA level (Supplementary Fig. 10b). The findings from the qRT-PCR analysis were further established at the protein level by immunostaining of induced neurons for MAP-2, & TH; and OLG for MBP (Supplementary Fig. 10c–e). The cells immune reactive to all the tested antibodies were present in the induced neuronal cells by day 6. However, no GFAP^+ve^ cells were observed upon immunostaining (data not shown). TH expressing cells indicate dopaminergic neurons. The ability of neurons and OPCs to co-survive in the same media was evident. The paracrine effects of neuronal cells and removal of GFs from the medium may have promoted the differentiation of OPCs to OLGs.

## Discussion

The multipotency of hADMSCs facilitates the derivation of different functional cells for regenerative applications. Stem-ness of the hADMSCs grown in culture in terms of proliferation and trilineage differentiation are important criteria for getting them committed to specific lineages as desirable. In this study, primary characterization using surface markers and trilineage differentiation confirmed the suitable quality of hADMSCs before attempting to derive NS. The neural cells derived from stem cells using artificial inducers often result only in transient changes with the upregulation of few neural markers. Exploiting the potential of GFs as inducers for stable neural differentiation of hADMSCs opens up several opportunities^14^. In the context of neural regeneration, conversion of mesodermal origin hADMSCs into stable ectodermal lineage is a major challenge.

The knowledge that the fibrin matrix supports the differentiation and proliferation of circulating NPCs to neurons^4,5^ prompted the design of cell-specific fibrin niche for differentiation of hADMSC to neural lineage cells. The highlight of the study is that the hADMSCs could be differentiated to different proliferating progenitor stages and then into neurons and OLGs. The behavior and characteristics of each cell type suggest the presence of a biomimetic process that makes these steady and stable changes in hADMSCs.

The Notch inhibitor has significantly blocked proliferation signals affecting NS formation. Seeding density and cell proliferation were found to be important for the transformation of plastic adherent hADMSCs to groups of cells within a short period of culture and developing into NPCs. The poor cell density resulting from the addition of chemical inhibitor of Notch confirmed that the proliferation signals are elicited through the Notch pathway. For the formation of NS, both proliferation and differentiation should occur simultaneously. Proliferating hADMSCs are contact inhibited and do not form spheres even when they undergo quick multiplication. In spite of poor Notch signals in the inhibited culture on INF, the hADMSC differentiated into Nestin^+ve^ NPCs and NPCs into OPCs expressing OLIG-2 and PDGFRα. However, proliferation was not sufficient for NS formation. Proving the differentiation, even in the absence of Notch, suggested an alternative mechanism for converting hADMSCs grown on fibrin matrix to NPCs and OPCs. It was proven that Wnt signals from fibrin are important for neural differentiation. The absence of inhibitor toxicity was established by MTT assay (Supplementary figure 11). Therefore, depletion of proliferation signal caused a reduction in cell numbers; not any cytotoxic action of the inhibitor.

The chemical inhibitor of Wnt signals also did not cause any cytotoxic effect on hADMSCs. However, the inhibitor affected proliferation, NS formation and NPC to OPC conversion. The stem cells treated with Wnt inhibitor did not differentiate to Nestin^+ve^ NS. Similarly, in the Wnt inhibited NPC culture on fibrin matrix, OPC-like cell morphology was rarely seen, OLIG-2 and PDGFα were downregulated as compared to that on INF, proving the role of Wnt in the differentiation of NPCs to OPCs which is achieved upon cell culture on a fibrin matrix. The presence of immunostained Wnt-3a in ECM of cells grown on INF and their absence upon adding chemical inhibitors prove the availability of Wnt signals in the fibrin niche. The presence of active β-catenin in the nucleus of cells grown on INF and its reduction upon the addition of chemical inhibitor reinforces the role of Wnt signaling in steps of hADMSCs to NPCs and NPCs to OPCs conversions.

The experiments using chemical inhibitors have proven that Notch signaling promotes hADMSC proliferation developing into NS, comprising NPCs, through the Wnt pathway. Thus, coordinated action of Notch and Wnt signaling in INF is the basis for NPC enriched NS formation. The dissociated NS also progressed into more mature and adherent proliferating NPCs expressing TUJ-1 and Nestin. These advanced NPCs differentiated into proliferating OPCs expressing OLIG-2 and PDGFRα through the Wnt pathway. Notch signaling elicited in INF did not influence differentiation; however, the proliferation of NPCs and OPCs were enhanced by this signal.

On the whole, the prominent role of the fibrin matrix in differentiating hADMSCs to NPCs and OPCs has been established. Fibrin plays the role of a cross-linking molecule for the retention of other adhesive proteins and GFs, which are the major components of the fibrin-based biomimetic niche. Both laminin and fibronectin present in the fibrin niche have been shown to support cell adhesion, proliferation and neural differentiation elicited by various signaling molecules^4,5^. In this study, the niche was modified with a different GF composition that converts the mesodermal cells to ectodermal cells before they are further differentiated to NPCs and in turn to glial cells and neurons. The EGF and bFGF used in the niche are associated with the trans-induction of hADMSCs into the ectodermal lineage. These GFs have also been reported to promote neural differentiation in higher doses^15^. Though morphologically, the NS appeared similar on TCPS and fibrin niche, the detachment with time indicates that proper signals for cell survival are absent on the bare plastic surface. This preliminary observation indicated that the use of GFs alone is not sufficient for producing stable NPCs and OPCs. Our study found promising results using a combination of adhesive proteins and different concentrations of GFs for differentiating hADMSCs to NS, NPCs, OPCs, neurons, and OLGs.

Apart from differentiation, the proliferation of hADMSC is also dependent on the signaling from its niche or microenvironment^16^. It has been reported that the NS comprising NSCs have self-renewal capacity and can be sub-cultured by mild trypsinization^17^. In our experiments, nearly 50% of the induced NS culture were Nestin positive. The Nestin^+ve^ NS were readily transformed to NPC lineage using induction media which contains a lower concentration of bFGF and EGF. The NPCs derived from NS in fibrin niche were proliferating progenitor cells with upregulated PCNA, TUJ-1, and NES expression. TUJ-1 marker is a more mature progenitor marker as it is associated with mitotically active neural progenitors and post-mitotic neurons^18^. The neurons developed from hADMSC-derived NPCs showed upregulated mature neuronal marker MAP-2 and NSE which agrees with the previous finding w.r.t Nestin^+ve^ circulating NPCs differentiation to neurons *in vitro*^19^. The addition of KCl into hADMSC-derived NPC culture helped in improving the neurite outgrowth and thereby achieving typical neuronal morphology as in the case of circulating NPCs^5^. Some of the induced cells were positive for dopaminergic neuron marker, TH. Both Wnt/β-catenin and FGF signaling pathways are key players in dopaminergic neurogenesis^20^. The soluble bFGF added to the niche would have obviously elicited the FGF signaling cascade enabling the differentiation of NPCs to dopaminergic neurons. In addition to that, a previous study using PBMNC-derived NPCs demonstrated Wnt-like neural inducing signals, establishing the role of the fibrin-based niche in neuronal differentiation by eliciting necessary signals^4^.

In physiology, both Wnt and Notch signaling molecules have an important role in glial differentiation. Canonical Wnt pathway or Wnt/β-catenin pathway is a key regulator of oligodendrocyte development^21^ and Notch signaling inhibits the neurogenin expression which promotes the up-regulation of OLIG-2 transcription factor^22^. Therefore, in the derivation of NS and OPCs from hADMSC in the presence of fibrin-based niche, the involvement of both Wnt and Notch signaling was explored and established. During active Wnt signaling, the cytosolic β-catenin is transported to the nucleus and by binding to the T-cell factor/lymphoid enhancer-binding factor, transcription of the Wnt target genes is initiated. The PNU 74654 binds beta-catenin and hinders its interaction with TCF/LEF and thereby inhibits the Wnt signaling^23^. The Notch receptors 1 to 4 are associated with a mammalian signaling system where the ligand binding to the receptors initiates the cleavage of the intracellular domain of receptor by γ-secretase. This Notch intracellular domain (NICD) translocates to the nucleus and activates the transcriptional Notch target genes. The Notch inhibitor, DAPT used in this study specifically inhibits the gamma-secretase enzyme component of the classical Notch signaling pathway there by continuing the inactivated state of the cells^24^.

The cells induced in the niche without inhibitor showed high cell to cell contact and cell density upon induction. A similar effect of DAPT or Notch signal inhibitor was seen in a human embryonic stem cell-based study, where neural rosette formation was negatively affected and promoted premature neural differentiation^25^. These results indicate the association of active Notch signals with higher levels of symmetric divisions and reduced death of neural stem cells, which results in rosette or sphere formation^25^. Wnt signaling also has a substantial role in regulating the symmetrical division of NSCs in the brain and it has been shown to promote neurosphere formation by NSCs *in vitro* and *in vivo*^26^. As compared to Notch, more prominent effect or downregulation was seen in Wnt inhibited cultures suggesting a greater role for Wnt signals in NS formation. The analysis of Notch downstream molecules using qRT-PCR revealed a reduced level of TCF-4 expression together with Hes-5 upregulation indicating active Notch signaling elicited in the fibrin niche. Ngn is a pro-neuronal molecule and its expression is inversely proportional to the upregulation of Notch signalling^27^. The dose-response of both chemical inhibitors were not systematically studied. Therefore, it is not clear from the study, if any one of the inhibitors used can cause complete inhibition.

In this study, a stage-wise slow differentiation is achieved using GF induction in the fibrin niche. During developmental neurogenesis, neurons and glial cells are derived from the same neural progenitors. Similarly, in this study, hADMSC derived NS progressed further to more mature NPCs that gave rise to both neural and glial cells. The major focus of this approach is to demonstrate stable differentiation of the same stem cells into different cell types of CNS, in a controlled biomimetic environment for translational purposes. The stage-wise differentiation protocol used for the induction of hADMSCs to OPCs and NPCs took nearly 15–16 days which is much less when compared to a previously published protocol for hADMSC trans-differentiation to OPCs which was 5 weeks long^13^. The number of GFs used is also relatively lesser than other widely used protocols.

In the context of CNS tissue regeneration, neuronal signaling plays an inevitable role in regulating the proliferation, differentiation, and survival of oligodendrocytes *in vivo*^28^. Therefore, a mixed population of OPCs and NPCs may be a good option for promoting interaction upon transplantation, facilitating better regeneration. In this study, it is observed that the biomimetic niche based on fibrin supported interaction of neurons and OPCs derived from hADMSCs *in vitro* cultures. It was observed that the co-culture of neurons and OPCs promoted differentiation of OPCs to OLGs in fibrin niche without any additional inducer. The immature and mature neuron markers, as well as OLG markers, were upregulated in the co-culture with no expression of GFAP marker. The results were consistent with the previous studies showing higher dopaminergic neuronal expression in soft fibrin gel-based culture system^29^.

The differentiation studies indicate that the fibrin-based matrix is a potent biomimetic niche for exploiting the multipotency of hADMSC for neural tissue engineering. However, some variability in marker expression was observed in different donors. The biomimetic niche incorporated with GFs enables stable differentiation of stem cells contrasting the transient chemical induction. Moreover, it is also a potent natural scaffold molecule that could be used as an injectable degradable cell delivery matrix for translational purpose^30^. The fibrin niche seemed to elicit signals for both proliferation and differentiation of hADMSC to NS.

The Hes-1 gene expression remained unchanged in the induced and inhibited culture. Similarly, the expression of Hes-1 protein was equally present in the nucleus of hADMSCs, inhibitor added cells and induced cells. This is suggestive of the presence of an active non-classical Notch signaling pathway which has a key role in *in vitro* differentiation of hADMSCs^31^. Moreover, the Hes-1 plays an important role in the interplay between β-catenin signaling and Notch classical signaling pathway^32^.

The requirement of biomimetic adhesive matrix-like poly-L-lysine/hyaluronic acid multilayer films has been identified for the successful conversion of neural stem cells to neurons^33^. A previous study established a fibrin-based niche eliciting biochemical signal directing the differentiation of circulating neural progenitors to neuron-like cells^5^. Apart from the differentiation of neural stem cells, the current study suggests a single fibrin composite matrix with different GF composition for generation stable Nestin^+ve^ progenitors and oligodendrocyte progenitors from mesenchymal stem cells in a stage-specific manner. Fibrin scaffold has a potential application in nerve regeneration because of its role in wound repair and regeneration^34^. This study advocates the potential application of fibrin niche as a cell delivery matrix in *in vivo* progenitor transplantation. Various studies show that fibrin promotes the regeneration, neuronal outgrowth or cellular migration when used as a scaffold for GF release or cell-based therapies for conditions like SCI^34–36^.

In summary, the defined seeding density of hADMSCs produces NS on a cell-specific fibrin-based composite niche, comprising insoluble adhesive proteins coated on tissue culture surface and soluble GFs added in the induction medium. The NS were dissociated and sub-cultured to produce more advanced NPCs expressing specific markers and with good proliferation potential. Through different steps and culture conditions, NPCs were differentiated into OPCs which were further differentiated into OLGs, expressing multiple marker proteins at each stage. Through yet another in-house standardized and published method, ADMSC-derived NPCs were also differentiated into neurons expressing multiple marker proteins. Electrophysiological studies were not carried out; however, demonstrated expressions of 4 different markers confirms the potential of the ADMSC-derived neuronal progenitor progression into mature neurons.

For all different stages and conditions standardized for obtaining cells of the central nervous system (CNS), the presence of fibrin matrix was found enhancing (i) hADMSC to NS (ii) NS to advanced NPCs; (iii) NPCs to OPCs; and (iv) NPCs to neurons. Specific roles of fibrin niche-based Notch and Wnt signaling pathways were established in both hADMSC to NS; and NPC to OPC conversions for achieving proliferating progenitors. The study established that by employing fibrin-based niche, hADMSCs can be transformed to proliferating progenitors with differentiation potential, within a short period of 7 to 15 days; thus, advocating pre-differentiation of autologous mesenchymal stem cells for potential, successful regenerative applications.

## Materials and Methods

### Human ADMSC culture and characterization

Human lipoaspirate samples collected from patients undergoing cosmetic surgery was used for ADMSC isolations. The Institutional Ethics Committee approval was obtained from the Kerala Institute for Medical Sciences (KIMS) hospital, Trivandrum, India. Informed consent from each participant was obtained before sample collection (KIMS-IEC/18012018 dated 08-05-2018). All *in vitro* methods of isolation, culture, and differentiation were carried out after obtaining approvals from the Institutional Ethics Committee and Institutional committee for stem cell research of Sree Chitra Tirunal Institute for Medical Sciences and Technology, Trivandrum conforming to the national regulations controlled by Indian Council of Medical Research, Govt. of India, New Delhi. The reference numbers of study approval are SCT/IC-SCR/44/March2017 and IEC NO:SCT/IEC/1231/June 2018. The ADMSCs were isolated and were expanded in culture^37^. At 80–90% confluence, the cells were passaged using standard trypsinization protocol. The cells were characterized based on cell surface markers and trilineage differentiation potential. Procedures, reagents, culture medium, antibodies used for characterization, etc. are given in detail in the Supplementary File.

### Preparation of fibrin matrix-based culture environment

Fibrin matrix-based niche used for differentiation and signaling studies was prepared using a modified, well-established protocol^38^. Pharmacopoeia grade fibrin sealant (Drug controller approved) was used for coating tissue culture polystyrene (TCPS, NUNC, Roskilder, Denmark).

Fibrinogen and thrombin were reconstituted using sterile distilled water. Thrombin (5 IU) was poured on to TCPS in a minimal volume to cover the TCPS surfaces. Incubation was done at 37 °C for 30 minutes and the thrombin was completely poured off after the incubation period. The thrombin adsorbed surface was layered with a thin layer of diluted fibrinogen (2 mg/ml). The clot formed was stabilized by incubating the coated TCPS at 37 °C for 30 minutes. The plates were frozen overnight at −80 °C. The plates were lyophilized (Modulyo 4 K, Edwards, UK) and stored at 4 °C in a sterile environment.

### Induction to neurospheres

The ADMSCs were plated in Fibrin coated TCPS and bare TCPS at a density of 10,000 cells/cm^2^ in DMEM/F12 basal media, with 10% Foetal bovine serum (FBS) and 1% AB/AM. After 24 hours, the basal media supplemented with 1% FBS, 1% AB/AM, 20 ng/ml basic fibroblast growth factor (bFGF) and 20 ng/ml epidermal growth factor (EGF) was added to the cells in bare TCPS (INB) and in niche (INF) (Abbaszadeh *et al*.^39^). 10% FBS and 1% AB/AM supplemented basal media was added to the control group, the un-induced ADMSC in fibrin. The medium was changed every 4^th^ day. Cells were maintained for 7 days and culture was terminated for further analysis using ICC, qRT-PCR, and flow cytometry.

### Reseeding of NS for Secondary NS generation

#### Reseeding of manually picked NS

The NS in fibrin niche were manually picked using a 10 µl tip and reseeded into bare and fibrin coated TCPS. The basal media, DMEM F12 (with 1% FBS) supplemented with AB/AM was provided for 7 days. The culture was terminated for further analysis of molecular markers.

#### Reseeding of NS dissociated with trypsin

The NS induced in fibrin niche was dissociated used mild trypsinization by day 7. The NS derived cells were seeded on to bare TCPS and fibrin niche in the presence and absence of growth factors (10 ng/ml of EGF and bFGF) in DEMEM F12 (1% FBS) supplemented with AB/AM for 5–7 days.

### Neural and glial progenitor inductions

Adherent NS formed from ADMSCs in NS induction media were harvested and plated on fibrin coated and bare TCPS in DMEM/F12 basal medium supplemented with AB/AM. The seeding density was 5000 cells/cm^2^. The next day the medium was supplemented with 10 ng/ml EGF and 10 ng/ml bFGF and the induced cells were maintained for another 4 days for the proliferation of the NPCs.

The induced NPCs were further supplemented with 10 ng/ml PDGF-AA and bFGF in basal medium (1% FBS) for OPC induction. (Modified protocol published by Abbaszadeh *et al*.^39^**)**. The culture was terminated in another 4 days to evaluate the differentiation using ICC, qRT-PCR, and flow cytometry.

### Differentiation to neurons

For neuronal induction, induced NPC culture was provided with DMEM F12 basal media added with 1% FBS and 20 ng of bFGF for 4 days followed by the addition of KCL at a final concentration 25 mM in 1% FBS-DMEM F12, for 2 days (Modified protocol^4,5^**)**. Control group, un-induced ADMSC was maintained in bare TCPS in basal media with 10% FBS & AB/AM. Induced cells were maintained for 4 days and culture was terminated for further analysis using RT-PCR and ICC.

### Differentiation of OPCs to oligodendrocytes

OPCs were further induced to OLG lineage with tri-iodothyronine (T3) hormone (20 ng/ml) along with growth factor removal for 72 hours in bare TCPS and fibrin coated TCPS. The ADMSC grown in bare TCPS using basal DMEM F12 media with 10% FBS and AB/AM was the control group. The culture was terminated for further analysis after 48 hours using ICC, qRT-PCR, and flow cytometry.

### Co-culture of induced neurons and OPCs

ADMSCs were induced to neurons and OPCs in fibrin niche as per previously described protocols. The seeding density was 5000 cells/cm^2^. The OPCs were then trypsinized and were seeded on to induced neural cells in fibrin niche maintained in the KCL medium (25 mM). The cells were maintained for 48 h in KCL containing 1% DMEM F12 media. ADMSC grown in basal DMEM F12 media with 10% FBS in bare TCPS was used as control.

*In vitro* differentiation of ADMSC into NPCs, OPCs and OLGs in the fibrin-based bio-mimetic niche were confirmed by analyzing the expression of various lineage-specific markers at genetic and protein levels using molecular methods PCR and ICC (Primer details & Antibody details in Supplementary file Tables 1 & 2). During different levels of differentiation, the cell morphology was periodically analyzed using phase-contrast microscopy (DMIRB, Leica Microsystems, Wetzlar, Germany).

### Effect of Notch & Wnt inhibitor on NS induction

The ADMSCs were plated in Fibrin coated TCPS and bare TCPS at a density of 10,000 cells/cm² in (DMEM/F12 medium, with 10% FBS and 1% AB/AM). After 24 hours the basal media (with 1% FBS, AB/AM) supplemented with 20 ng/ml basic fibroblast growth factor, 20 ng/ml epidermal growth factor and either one of the inhibitors. Two concentration of inhibitors were used, 20 µM/ml (LC); 40 µM/ml (HC)- Wnt inhibitor (PNU 74654, Tocris) and 10 µM/ml (LC); 20 µM/ml (HC)- Notch inhibitor (DAPT, Abcam). Induction media (20 ng/ml bFGF & EGF, 1% FBS, AB/AM) was added to cells in fibrin coated TCPS (INF). In cells seeded in bare TCPS, 10% FBS supplemented DMEM f12 medium was added (Control group). Medium change was done on every 4^th^ day.

### Effect of Notch & Wnt inhibitor on OPC induction

NS derived cells were induced to NPCs in fibrin coated TCPS and were supplemented with OPC IM (10 ng/ml bFGF, 20 ng/ml PDGF-AA) and either one the inhibitors. Two concentration of inhibitors were used, 20 µM/ml (LC) and 40 µM/ml- Wnt inhibitor (HC); 10 µM/ml (LC) and 20 µM/ml (HC)- Notch inhibitor (DAPT). In cells seeded in bare TCPS, 10% FBS, AB/AM supplemented DMEM F12 medium was added (Control group). The culture was terminated for further analysis on day 4.

Following each stage of induction, gene expression of stage-specific molecular markers was estimated. The total RNA from cells were collected using TRIZOL reagent (Invitrogen, USA) based on the manufacturer’s protocol. RNA quantification was done using spectrophotometry in a Nanodrop equipment (ND 2000; Thermo Scientific, USA). After quantification, 200 ng of total RNA was converted to cDNA using the Orion XcDNA kit (Origin, India) using a thermal cycler (Master cycler; Eppendorf).

Stage-specific gene expression of the induced cells after induction time period was analyzed using qRT-PCR, ICC and flow cytometry (Primer details & Antibody details in Supplementary file Tables 1 & 2).

Real-time PCR was carried out in a 40x reaction using the Bio-Rad iQ5 (Reaction mix: 20 ng cDNA, 100 pmol each of specific forward and reverse primers,7.5 µl of OrionX 2X Real-time PCR master mix (Origin, India)). GAPDH was used as the house-keeping gene. The variation in gene expression was calculated as fold change after normalization with GAPDH expression using the formula 2^−ΔΔCt^.

For ICC the cells were fixed using 3.7% formaldehyde for 20 minutes. The cells were then washed with PBS and permeated using 0.1% Triton-X for 5 minutes for all nuclear and cytoplasmic proteins. For reducing non-specific binding, 3% BSA in PBST for 30 minutes was used for blocking. Then cells were incubated overnight at 4 °C with the standardized dilution of primary antibodies. Following the incubation, cells were washed with PBS and further stained with specific secondary antibodies. After 1 h incubation at room temperature, the cells were washed thrice with PBS for 5 minutes and were counterstained with nuclear stain, DAPI. The fluorescence was observed using fluorescent microscope (Leica microsystems, DMIRB, Germany).

For flow cytometry the cells were fixed and stained using the protocol similar to ICC. Finally, the cells were resuspended with the 500 µl of PBS. Unstained cells were used for gating the cells in the flow cytometer (Cytoflex, Beckman Coulter, Germany). For primary antibodies conjugated with fluorochrome, unstained was used as the control. And for the secondary antibodies conjugated, cells incubated with secondary antibody alone used as the control. The data was analyzed using FlowJo and CytExpert software.

Western blotting was done using total cell lysate from Control ADMSCs on bare TCPS, NPCs and OPCs induced on fibrin. The total protein was isolated using RIPA buffer added with proteinase inhibitor. 8% and 10% SDS gel was used for Nestin and OLIG-2 markers respectively. 60 µg protein was loaded in each lane and the separated proteins were blotted to the PVDF membrane using a semi-dry western blotting system (0.1 mA/cm^2^ & 25 V; 1 h for Nestin and 30 minutes for OLIG-2; Bio-Rad turbo Transblot system; Bio-Rad USA). The blots were blocked using 5% BSA for 1 h at room temperature and incubated in primary antibody (1:1000) overnight at 4 °C. Then the blots were incubated with HRP conjugated secondary antibodies for 1 h at room temperature. PBST wash was given thrice for 5 minutes after primary and secondary antibody incubation. The bands were visualized using developing solution (0.05 % diaminobenzidine (DAB), 0.04 % nickel chloride and 0.1% hydrogen peroxide in PBS).

### Quantification of cell fluorescence using imageJ

The ImageJ software (NIH) was used to measure the area, integrated density and mean gray values of selected cells in the ICC images (40X magnification; (MAP-2, Syn & NGFR of induced neurons; TH & OSP in co-culture). The cells of interest were marked using the drawing tool and the area, integrated density and mean gray value was measured. Corresponding background values were also measured. The fluorescence measure of cells was calculated as corrected total cell fluorescence (CTCF) using the equation, CTCF = Integrated density − (Area of the selected cell X Mean fluorescence of background readings)^40^ and represented as histograms. Average CTCF of 15 cells from 5 or more fields were used for accuracy.

### Statistical analysis

Statistical significance was calculated by ANOVA (single factor) for all quantitative data having more than two groups. Students t-test was used for comparing two groups. Mean and standard errors (SEM) were calculated for all parameters and are represented in graphs. Significance is labeled in the graphs with ‘***’ (P < 0.001); ‘**’ (P < 0.01); and ‘*’ (P < 0.05).

### Ethical approval & Informed consent

All authors have agreed to be co-authors. Communication with your esteemed journal has been approved formally by the Head of our Institute. Approval copy is available for submission if required.

Informed consent was taken from the donors who agreed to provide the lipoaspirates which is a requirement for obtaining IEC/IC-SCR approvals and was taken from time to time. For the collection of lipoaspirates, the Institutional ethics committee (IEC) of KIMS hospital, Trivandrum India has reviewed a proposal submitted by Dr. Manesh Senan and discussed during a meeting held on 18^th^ Jan 2018 and approval was issued. Also, for carrying out research using hADMSCs isolated from lipoaspirate collected at KIMS hospital, the IEC of SCTIMST and stem cell committee of SCTIMST approvals were obtained. The reference numbers of study approvals obtained from SCTIMST are SCT/IC-SCR/44/March2017 and IEC NO:SCT/IEC/1231/June 2018. All approvals from KIMS and SCTIMST are linked to the guidelines stipulated by Indian Council for Medical Research, Govt. of India.

## Supplementary information

Supplementary information.
