# Supplementary material for: Exploitation of fibrin-based signaling niche for deriving progenitors from human adipose-derived mesenchymal stem cells towards potential neural engineering applications
Source: Sci Rep. 2020 Apr 28;10:7116. doi: 10.1038/s41598-020-63445-2 (PMC7188903; doi:10.1038/s41598-020-63445-2)
Supplement: Supplementary file 1 — Supplementary information. [file 41598_2020_63445_MOESM1_ESM.pdf]

SUPPLEMENTARY FILE

Exploitation of fibrin-based signaling niche for deriving progenitors from human adipose-derived mesenchymal stem cells towards potential neural engineering applications

Authors: Krishnapriya Chandrababu M. Sc[1], Manesh Senan, MCh[2]; Lissy K Krishnan, PhD[1*]

E-mail: lissykrs@gmail.com ; bio.krishnapriya@gmail.com; maneshsenan@yahoo.com

Characterization of ADMSCs

hADMSCs from passage 2-4 were used for characterization using surface markers as listed in supplementary table1, and confirmed by tri-lineage differentiation and specific staining.

Cocktails of fluorochrome-labeled antibodies were used for staining MSCs. A cocktail with MSC positive markers, CD73 (APC), CD90 (FITC), CD105 (PE)) and MSC negative markers, CD45, CD34, CD14, CD 20 (PerCP) was used to stain passage 3 ADMSCs. The cocktail and the isolated cells were mixed well followed by a 10 minutes incubation at 4ºC. Fluorochrome labeled antibody in another cocktail was used as isotype control (PerCP, PE, APC, FITC). The analysis was done using flow cytometer (Beckman Coulter, Germany). CytExpert software was used for data analysis.

The StemPro Adipogenesis differentiation kit (Invitrogen), StemPro Chondrogenesis differentiation kit (Invitrogen) and the StemPro Osteogenesis differentiation kit (Invitrogen) were used to induce differentiation of MSCs into adipogenic, chondrogenic and osteogenic tissues, respectively according to the manufacturer's instructions.

Passage 3 (P3) hADMSCs were seeded at a density of 10,000 cells/cm^2 and cultured in low glucose DMEM, 10% FBS and 1% AB/AM solution for 24 hours. The medium was then replaced with adipogenesis differentiation medium and the cells were incubated at 37˚C with 5% CO_2. The differentiation medium was replenished every third day for an induction period of 21 days. After induction, the cells were fixed with 3.7% formaldehyde, rinsed with PBS and the stained with specific stain Oil Red O staining to confirm differentiation.

P3 hADMSCs were seeded at a density of 10,000 cells/cm^2 in DMEM low glucose medium with, 10% FBS and 1% AB/AM. The medium was replaced with osteogenesis differentiation medium after 24 h. and was incubated at 37˚C with 5% CO_2. Every third day the differentiation medium was replenished till an induction period of 21 days. Post induction, the cells were fixed with 3.7% formaldehyde, rinsed with PBS and stained with specific stain Alizarin Red to confirm the differentiation.

P3 hADMSCs were seeded at a density of 5,000 cells/cm^2 in basal growth medium for 24 hours. Chondrogenic differentiation medium was supplemented thereafter and the cells were incubated at 37˚C with 5% CO_2. The medium change was done every third day for an induction period of 21 days. After induction, the cells were fixed with 3.7% formaldehyde, rinsed with PBS and stained with specific stain Toluidine blue to confirm the differentiation.

Proof of stem-ness: The hADMSC grown in culture were spindle shaped and plastic adherent and they maintained these properties till 10-14 passages. The cells showed the potential to differentiate into adipogenic, osteogenic and chondrogenic lineages and the multipotency of ADMSCs was confirmed using special stains. Oil Red O staining revealed the presence of lipoid substances confirming adipogenic differentiation of hADMSC, Alizarin Red Staining detected the presence of calcium precipitate formed in osteo-lineage committed cells and Toluidine blue staining identified the presence of acidic proteoglycan present in cells committed to chondrogenic differentiation (Supplementary figure 1 a-c). Flow cytometric analysis of stem cell markers CD105, CD73 & CD90 showed ~95% of the cells in culture to be positive. The markers of hematopoietic stem cells (HSCs) CD14 & CD45 showed < 5% positivity for the same population (supplementary figure 1 d-g).

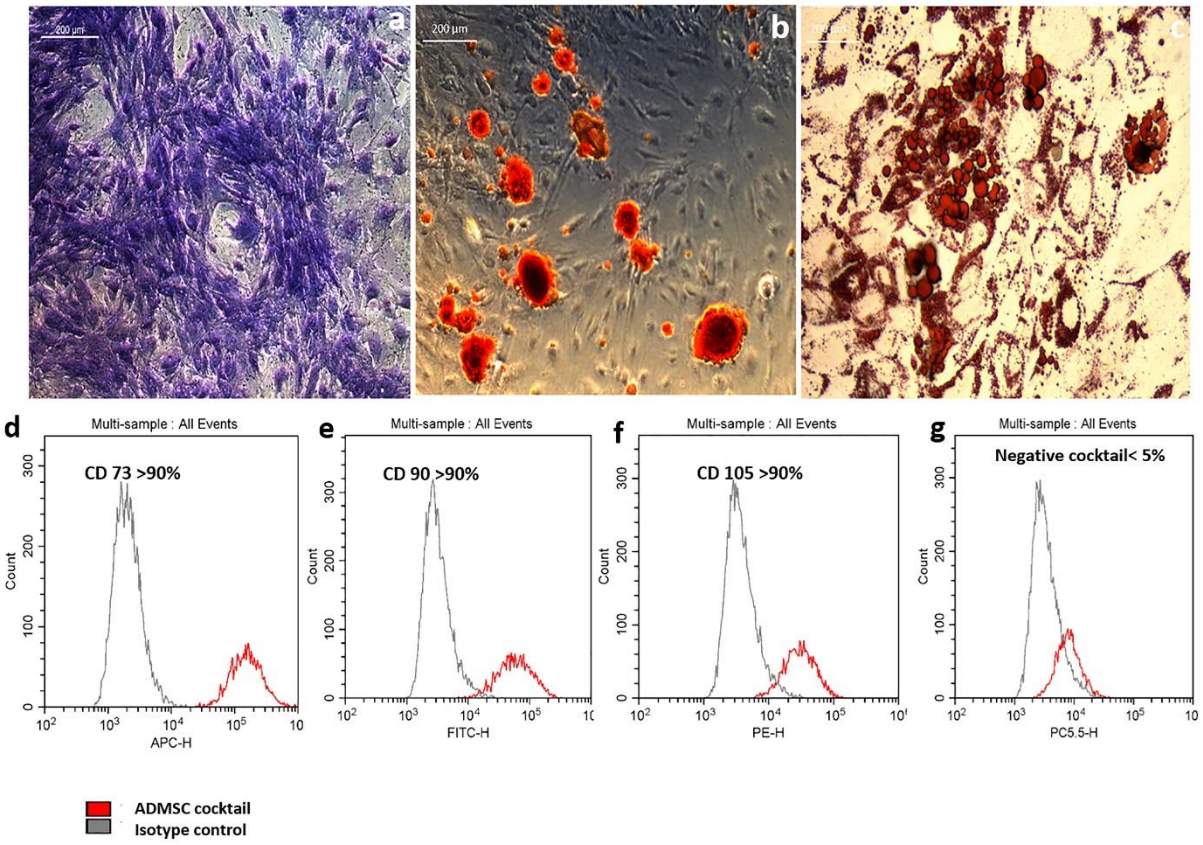

Supplementary figure 1. Characterization of hADMSCs: Micrographs depicting trilineage differentiation (a) Toluidine blue staining of acidic proteoglycan in induced chondrocytes (b) Alizarin Red Staining of the calcium precipitate in induced osteocytes (c) Oil red-O staining of lipoid droplets in induced adipocytes. Histograms showing flowcytometric analysis of hADMSCs for a panel of positive and negative CD markers (d) CD 73 (e) CD 90 (f) CD 105 (g) Histogram representing negative cocktail consist of markers CD 14, CD 34 and CD 45.

Proof of signaling mechanism in hADMSC to NS conversion in INF:

The abundance of immunostained wnt-3a molecules in cytoplasm and ECM of cells in INF and their absence in inhibited-INF is presented proving the effect of inhibitors. The effect of inhibitors in differentiation is presented in fig 3 in the main text.

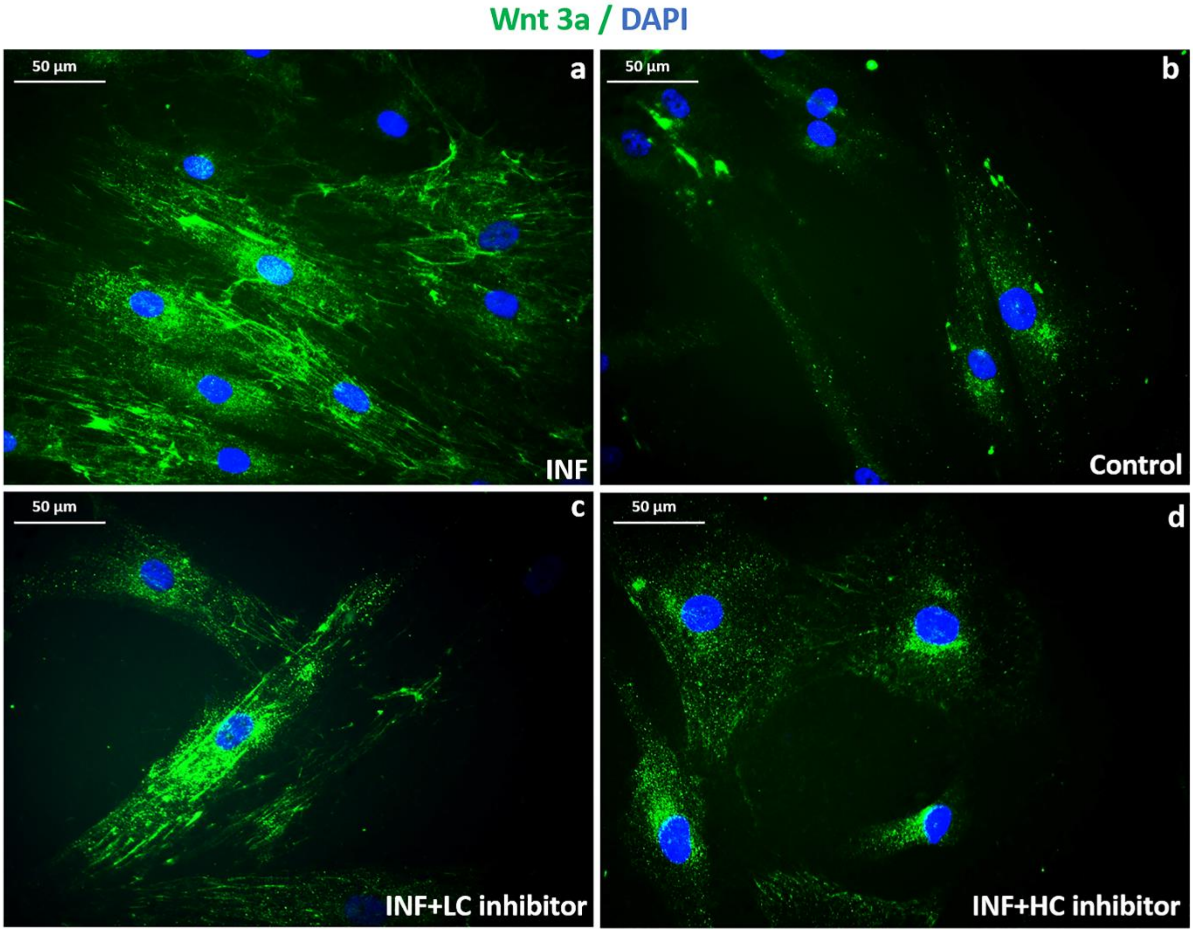

Supplementary figure 2. Fluorescence micrograph of cells immunostained with wnt 3a antibody: (a) Cells induced to NS expressing wnt 3a molecules in cytoplasm and ECM in control niche-INF; (b) hADMSC control grown in bare TCPS showing no positive signals in ECM; (c) wnt-3a (LC; 20 μM/ml) inhibited INF cells with infrequently stained wnt-3a in ECM; (d) wnt-3a (HC; 40 μM/ml) seen only in cytoplasm of few cells. DAPI used as nuclear stain.

The abundance of immunostained β-catenin molecules in the cytoplasm and nucleus of cells in INF and their absence in inhibited-INF is presented proving the effect of inhibitors. The effect of inhibitors in differentiation is presented in fig 3 in the main text.

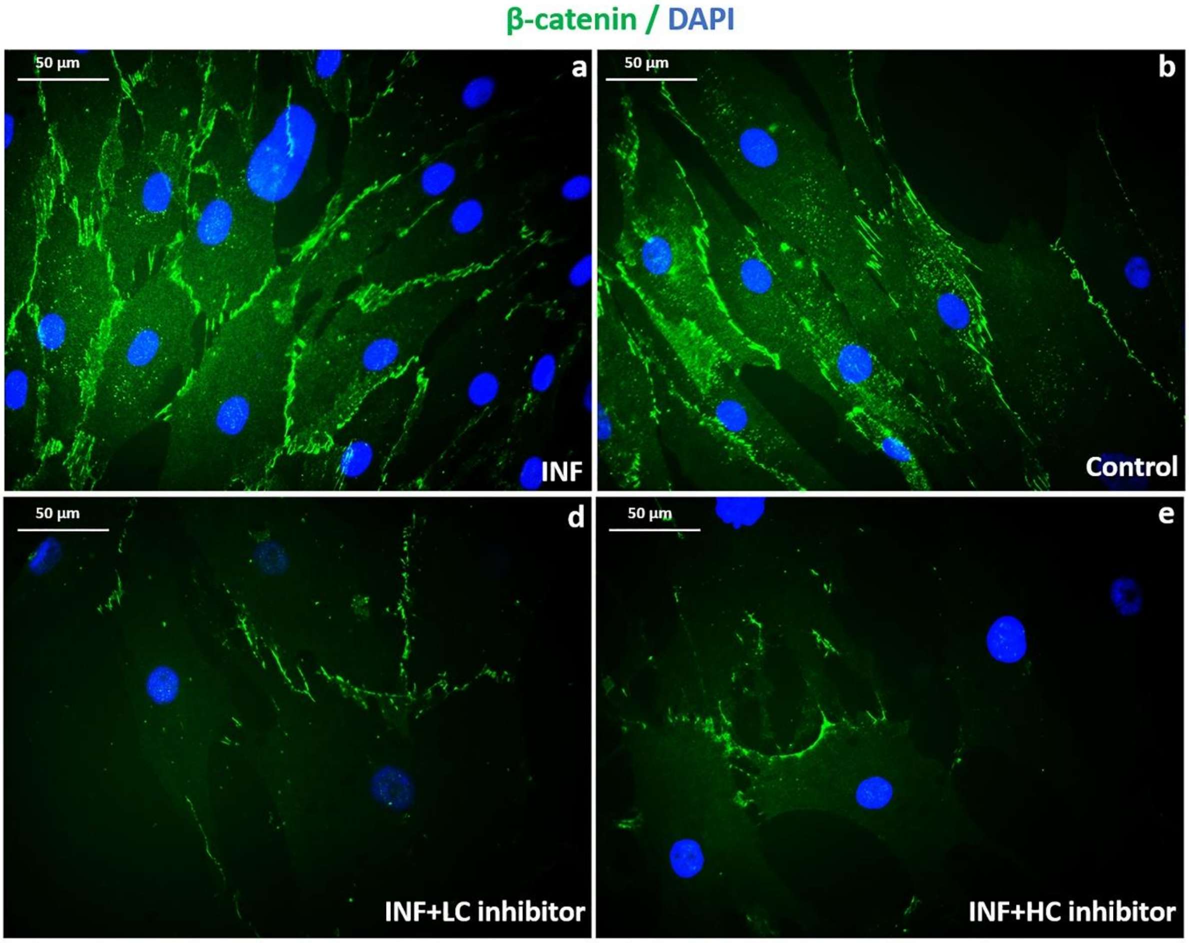

Supplementary figure 3. Fluorescence micrograph of cells immunostained with Beta catenin antibody: (a) Cells induced to NS in INF expressing beta-catenin in cytoplasm and nucleus; (b) hADMSC control in bare TCPS with stained β-catenin in cytoplasm and rarely in nucleus; (c) wnt-3a inhibited (20 µM/ml) INF cells showing very few cells positively stained in cytoplasm and nucleus; (d) wnt-3a inhibited (40 µM/ml) INF cells showing hardly any cells positively stained in cytoplasm and nucleus. DAPI used as nuclear stain.

The abundance of immunostained notch molecules in the cell nucleus upon growing in INF and their absence in inhibited-INF is presented proving the effect of inhibitors. The effect of inhibitors in differentiation is presented in fig 3 in the main text.

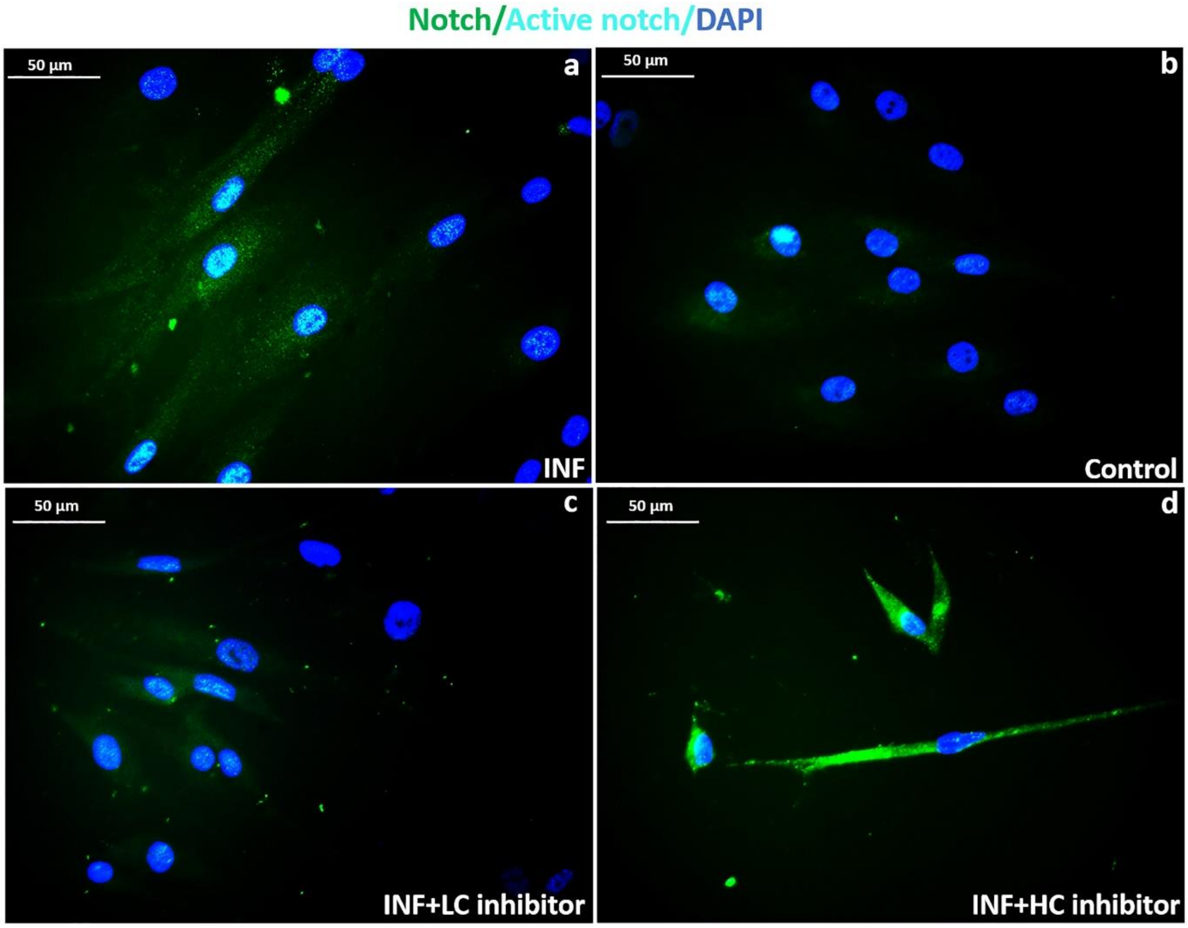

Supplementary figure 4. Fluorescence micrograph of cells immunostained with Notch antibody: (a) Cells induced to NS in INF; (b) hADMSC control in INB; (c)Niche added with low concentration of inhibitor (LC; 10 µM/ml); (d) Niche added with higher concentration of inhibitor (HC; 20 µM/ml). DAPI used as nuclear stain. Notch molecules located in the nucleus shows cyan color.

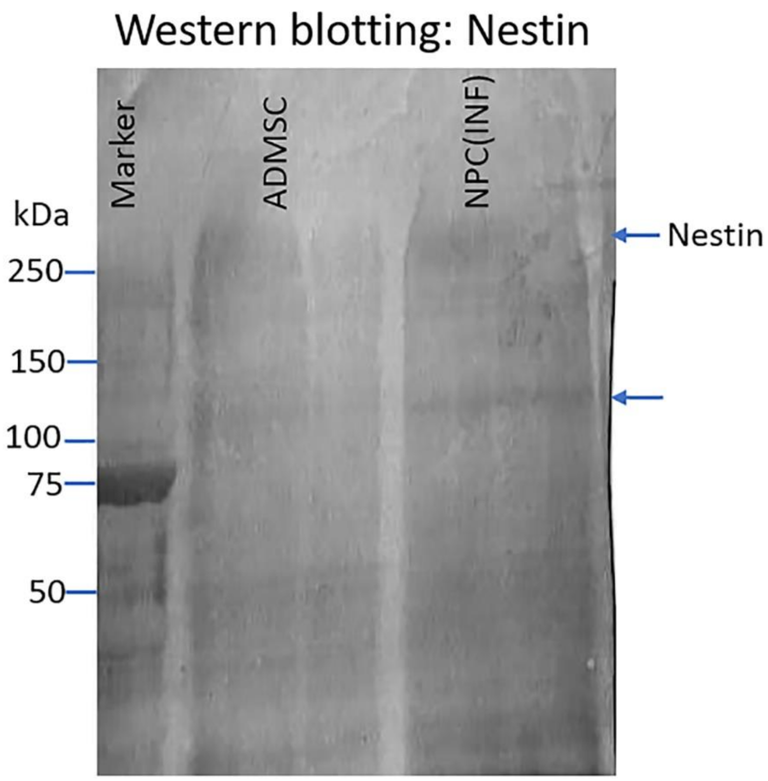

Supplementary figure 5: Western blotting analysis of NPCs: Band corresponding to Nestin was observed at ~ 260 kDa and ~120 kDa**.**

Analysis of of OPCs in culture using OLIG 2 as marker

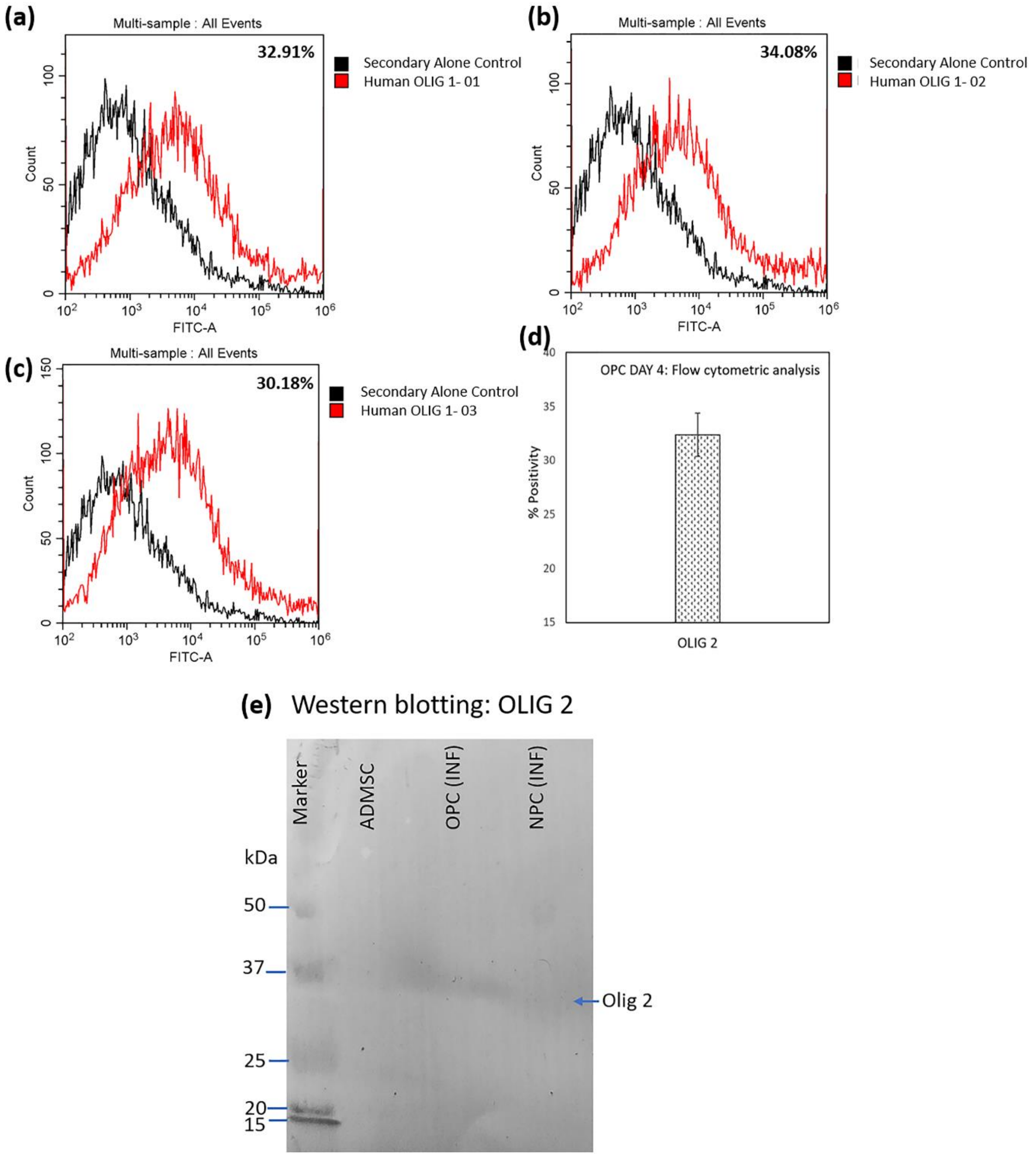

Supplementary figure 6. Flowcytometric histogram of induced OPCs using OLIG 2 positive population: The histograms (CytExpert software) representing OLIG 2 positivity in induced OPCs in INF: (a) Donor 1; (b) Donor 2; (c) Donor 3; (d) Graphical representation of compiled data(Avg ±S.D., n=3). Secondary antibody alone stained induced cells used as the control for setting gate position (e) Western blot analysis of OLIG 2: The corresponding band was observed at ~36 kDa ADMSCs grown on bare TCPS and NPCs induced on fibrin used as control.

Proof of signaling mechanism in NPCs to OPC conversion in INF

The abundance of immunostained notch molecules in cell nucleus upon growing in INF and their absence in inhibited-INF is presented proving the effect of inhibitors. The effect of inhibitors in differentiation is presented in fig 6 in the main text.

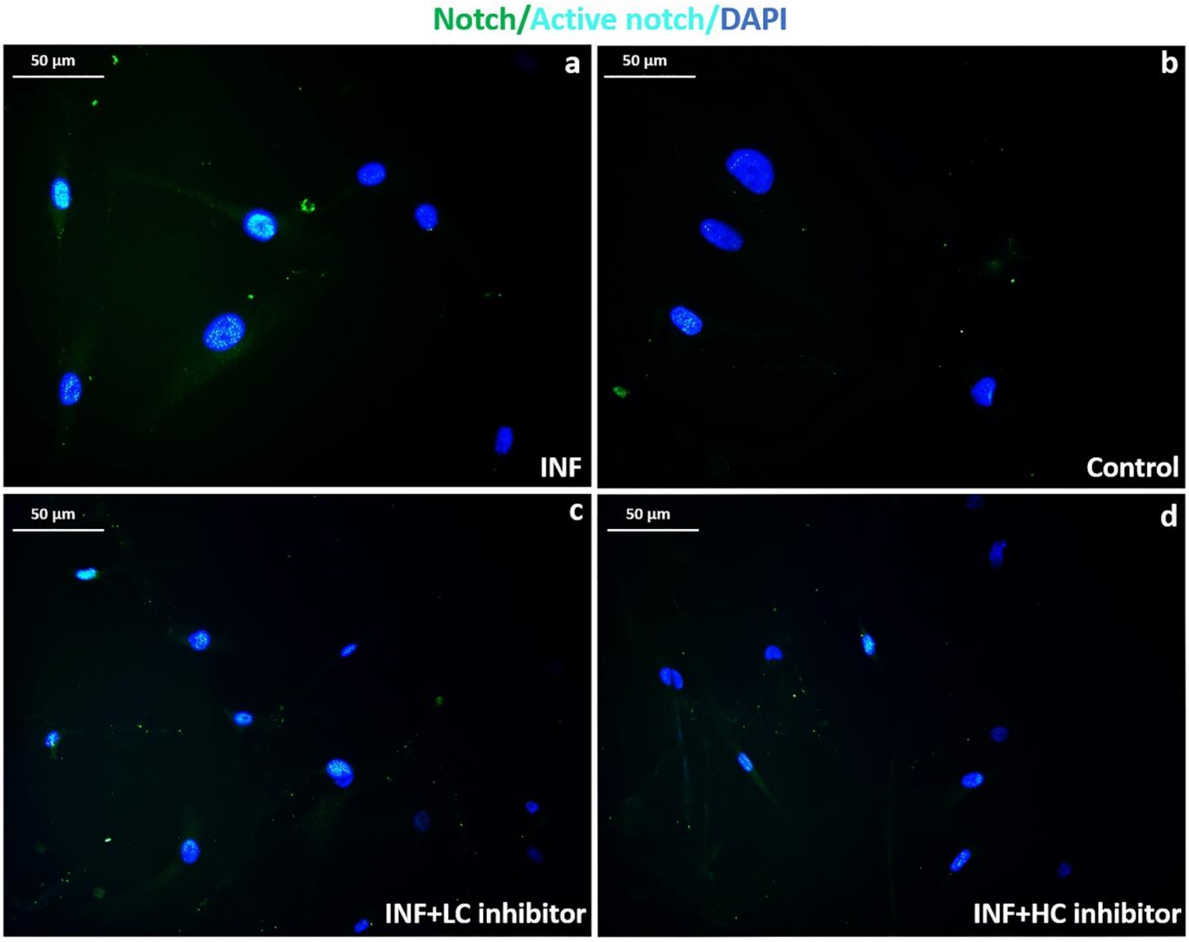

Supplementary figure 7. Fluorescence micrograph of OPC immunostained with Notch antibody: (a) Cells induced to NS in INF; (b) hADMSC control in INB; (c)Niche added with low concentration of inhibitor (LC; 10 µM/ml); (d) Niche added with higher concentration of inhibitor (HC; 20 µM/ml). DAPI used as nuclear stain. Notch molecules located in the nucleus shows cyan color.

The abundance of immunostained wnt-3a molecules in cytoplasm and ECM of cells in INF and their absence in inhibited-INF is presented proving the effect of inhibitors. The effect of inhibitors in differentiation is presented in fig 6 in the main text.

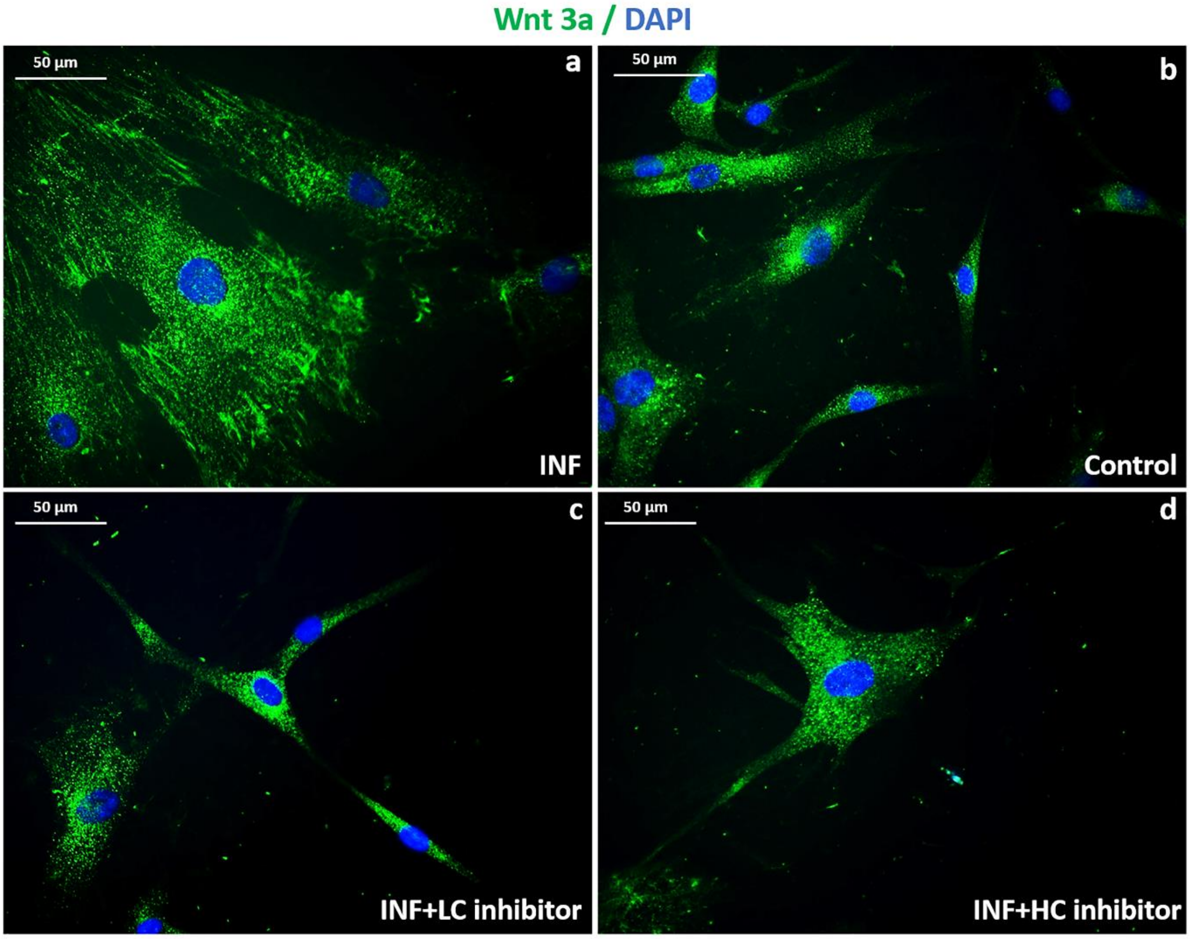

Supplementary figure 8. Fluorescence micrograph of OPCs immunostained with wnt 3a antibody: (a) Cells induced to NS expressing wnt 3a molecules in cytoplasm and ECM in control niche-INF; (b) hADMSC control grown in bare TCPS showing no positive signals in ECM; (c) wnt-3a (LC; 20 μM/ml) inhibited INF cells with infrequently stained wnt-3a in ECM; (d) wnt-3a (HC; 40 μM/ml) seen only in cytoplasm of few cells. DAPI used as nuclear stain.

The abundance of immunostained β-catenin molecules in the cytoplasm and nucleus of cells in INF and their absence in inhibited-INF is presented proving the effect of inhibitors. The effect of inhibitors in differentiation is presented in fig 6 in the main text.

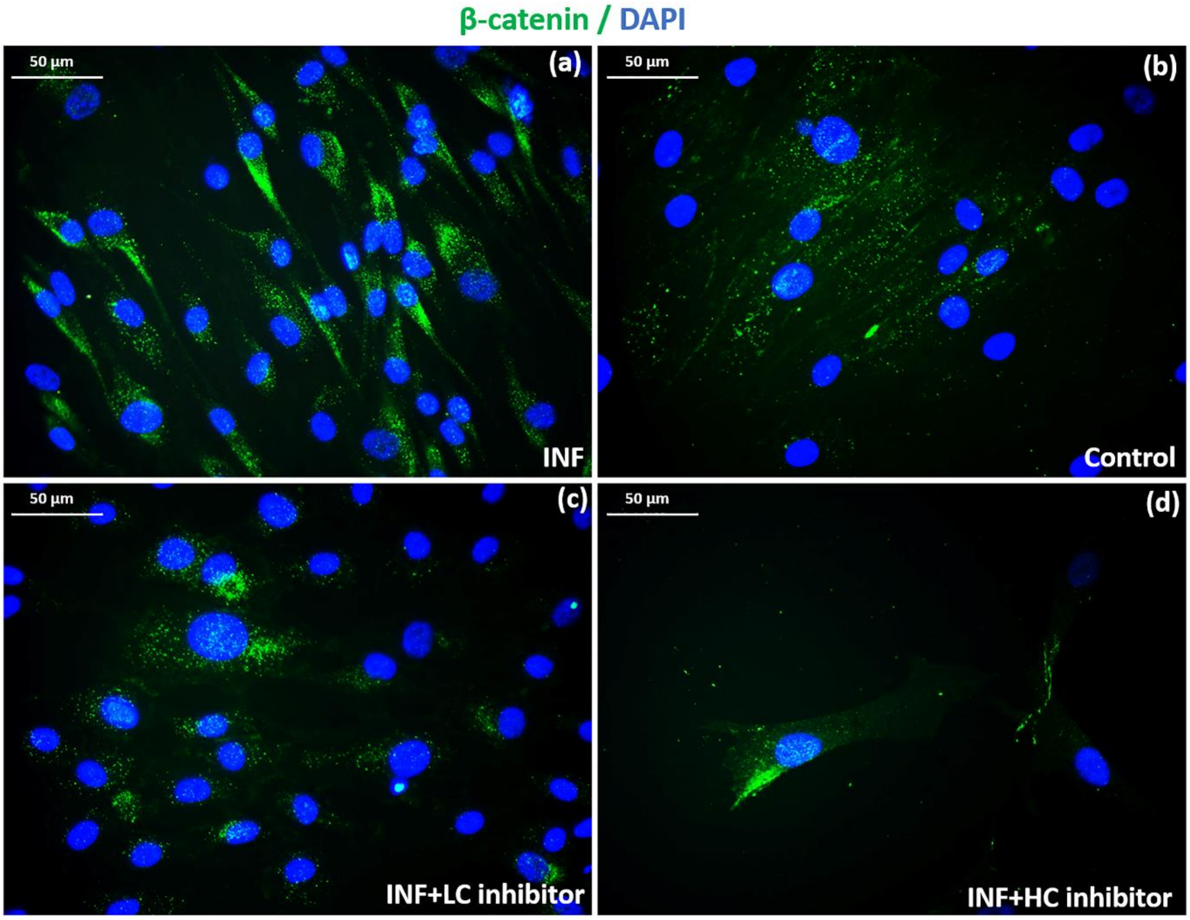

Supplementary figure 9. Fluorescence micrograph of OPCs immunostained with Beta catenin antibody: (a) Cells induced to NS in INF expressing beta-catenin in cytoplasm and nucleus; (b) hADMSC control in bare TCPS with stained β-catenin in cytoplasm and rarely in nucleus; (c) wnt-3a inhibited (20 µM/ml) INF cells showing very few cells positively stained in cytoplasm and nucleus; (d) wnt-3a inhibited (40 µM/ml) INF cells showing hardly any cells positively stained in cytoplasm and nucleus. DAPI used as nuclear stain.

Co-culture & Survival of neurons and OPCs:

Proof of co-survival of neurons and OPCs at m-RNA level with upregulated markers of both cell phenotypes and at protein level seen in immunostained cells detecting positive markers for both cell types is presented.

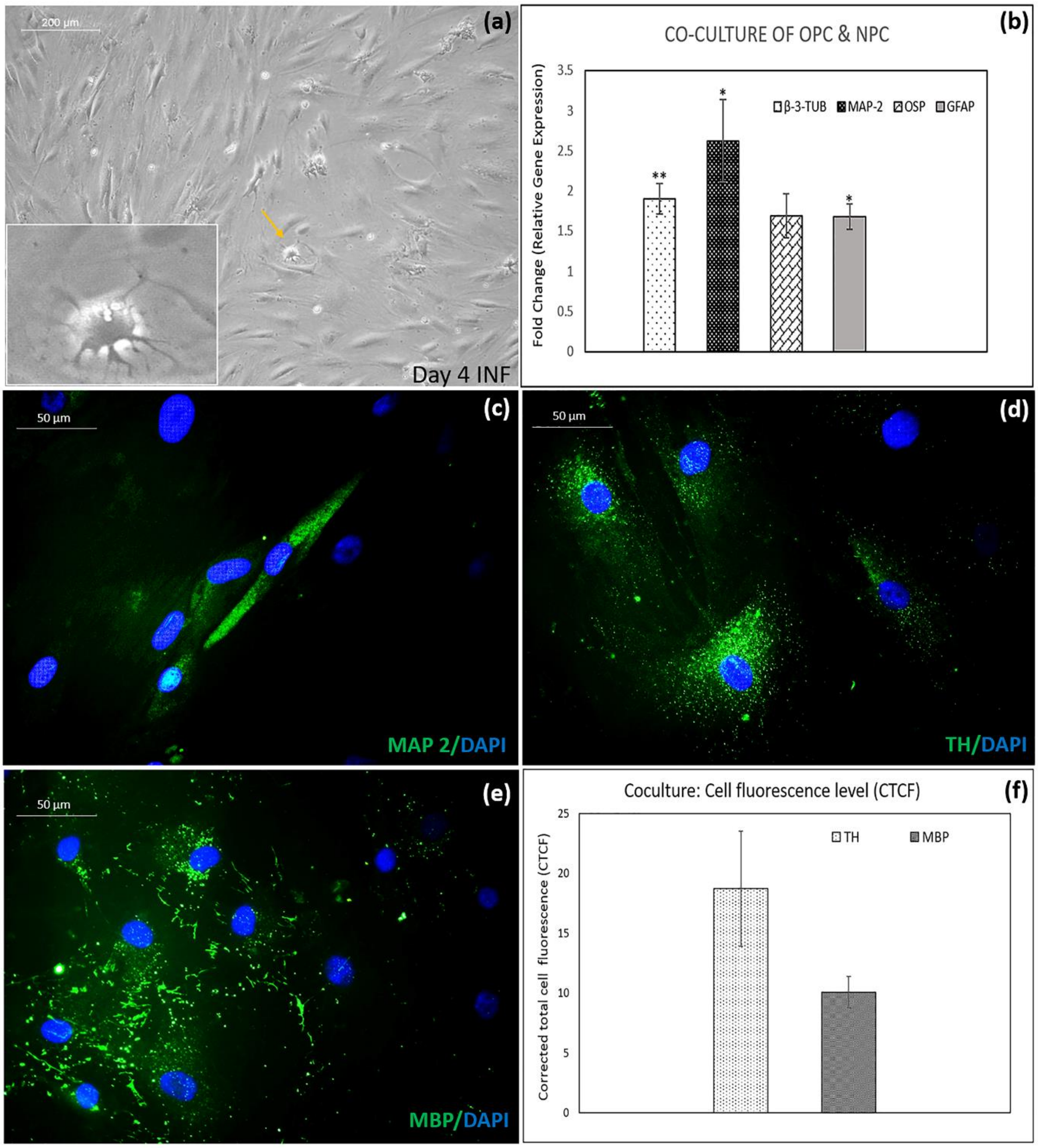

Supplementary Figure 10. Coculture of OPCs and neurons (a) Phase contrast micrograph showing the morphology of co-cultured neurons and OPCs. Higher magnification of a cell with OLG morphology is in the inset (Region of interest in Yellow arrow); (b) Graphical representation of qRT-PCR analysis of neuronal and glial markers in the co-culture; TUJ 1, MAP-2, OSP and GFAP gene expression compared to control expressed as individual bars. TUJ I (P=0.009), MAP-2 (P=0.05) and GFAP (P=0.04) - hADMSC in bare TCPS in DMEM F12 media was used as the experimental control; GAPDH was used as the Housekeeping gene; Student's t-test:

Control & INF; ('***' ($P \leq 0.001$), '**' ($P \leq 0.01$), '*' $P \leq 0.05$)); Error bars represent SEM. Fluorescence micrographs shown are (c) MAP2; (d) TH ; (e) MBP immune positive cells in the coculture. DAPI used as nuclear stain. (f) Quantitative fluorescence image analysis: Corrected total cell fluorescence (CTCF) calculated from ICC images of TH and MBP (Average of CTCF of 15 cells from 5 fields). Error bars represent SEM.

MTT assay: Use of chemical inhibitors to establish signaling mechanism

The human ADMSCs grown in fibrin niche at a seeding density of 5000 cells /cm ^{2} were used for MTT assay. The inhibitors were added to the ADMSCs for 48 h. The wnt inhibitor and notch inhibitor was added at a concentration of 40µg/ml. The cells were washed with sterile HBSS and the MTT reagent (Methylthiazolyldiphenyl-tetrazolium bromide, Sigma Chemicals-USA) was added to the culture and was incubated for 2 h. MTT lysis buffer, DMSO (100 µL) was added and the incubation continued for an additional 4 h. The suspension was transferred to a 95 well plate and the absorbance was measured at 570 nm. The percentage of viable cells as compared to control in culture was calculated using the formulae: Absorbance of Test X Absorbance of Control/100 [31].

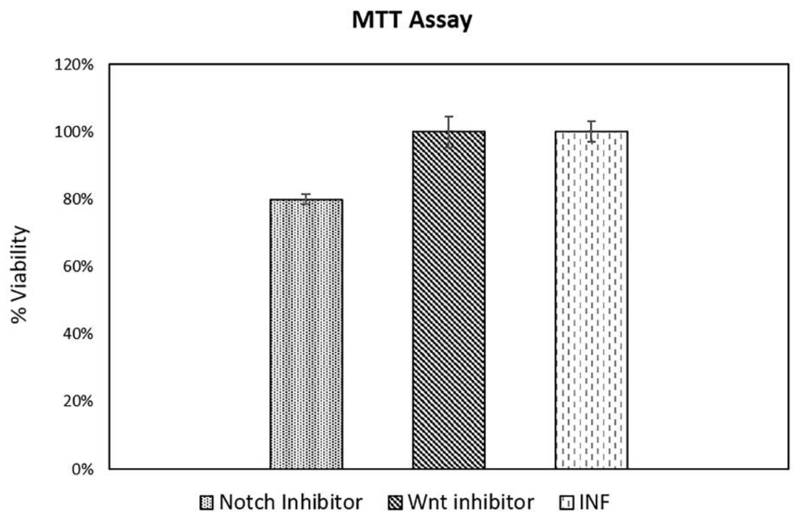

Supplementary figure 11: Graphical representation MTT assay. Cell viability, 48 h after addition of inhibitor to the ADMSC culture in INF. Error bar represents SEM. ADMSC in neural induction niche without addition of inhibitor was used as control.

Table 1: List of primers and their sequences

Sequence Definition	**Sense Primer**	**Anti-sense Primer**
GAPDH	GCTTGTCATCAATGGAAATCCC	TCCACACCCATGACGAACATG
Nestin	GCCCTGACCACTCCAGTTTA	GGAGTCCTGGATTTCCTTCC
TUJ 1	GCTCAGGGGCCTTTGGACATCTCTT	TTTTCACACTCCTTCCGCACCACATC
PDGFRα	AGGTTGAGAGGAGGACTT	CCACTGAGATGCTACTGAG
OSP	ACTGCTGCTGACTGTTCTTC	GTAGAACGGTTTTCACCAA
MBP	GATGGCGTCACAGAAGAG	CCGATGGAGTCAAGGATG
PCNA	GGCCGAAGATAACGCGGATAC	GGCATATACGTGCAAATTCACCA
Hes 1	CGTCTACACCAGCAACAG	TCCTCTTCTCTCCCAGTATTC
Hes 5	ATCCTGGAGATGGCTGTC	CTTCGCTGTAGTCCTGGT
TCF4	CCAGACTACTCCGTTCCT	AACACCAACAACAACAAGAAG
NGN 2	AAGAGATGATGGTGGCATATC	CAAGTCCTTCGGCGTTAA

Table II : List of Antibodies and their sources

**Antibody**	**Details**
**ADMSC characterization** (CD73 (APC), CD90 (FITC), CD105 (PE); CD45, CD34, CD14, CD 20 (PerCP)	Miltenyi Biotech MSC phenotyping kit
Nestin (1:100), O4 (1:200), synaptophysin (1:100)	R& D systems, Minnepolis, USA
PCNA (1:500), TUJ 1(1:500), Olig 2 (1:1000), PDGFRα (1:1000), OSP (1:1000), MBP (1:500), Beta catenin (1:1000),	Abcam, UK

Anti- notch 1 (1:500), Anti-wnt 3a (1:500), TH (1:500), NGFR (1:500)	
Nuclear stain: 4,6-diamidino-2-phenylindole (DAPI; 1:5000)	Invitrogen, USA.
GFAP (1:500)	BD Biosciences, India
MAP-2 (1:100)	Santa Cruz, Heidelberg, Germany
